# Improving autotrophic D-lactate production by heterologous expression of a pyruvate formate lyase in an LDHD-expressing *Acetobacterium woodii* strain

**DOI:** 10.1007/s00253-026-13952-5

**Published:** 2026-07-10

**Authors:** Kira S. Baur, Anna Stock, Dirk Weuster-Botz, Frank R. Bengelsdorf

**Affiliations:** 1 Institute of Molecular Biology and Biotechnology of the Prokaryotes, University of Ulm, Albert-Einstein-Allee 11, 89081 Ulm, Germany; 2 Chair of Biochemical Engineering, Technical University of Munich, Boltzmannstraße 15, 85748 Garching, Germany

**Keywords:** D-Lactate, Metabolic engineering, Anaerobic metabolism, *Acetobacterium woodii*, Gas fermentation

## Abstract

The anaerobic acetogen *Acetobacterium woodii* is a promising candidate producing chemicals while converting the greenhouse gas CO_2_. In this study, *A. woodii* was genetically engineered to express a lactate dehydrogenase (LDHD) from *Leuconostoc mesenteroides*, and a pyruvate formate lyase (PFL) as well as its activating enzyme (PFL-AE) originated from *Clostridium pasteurianum* to autotrophically produce D-lactate next to acetate, using H_2_ + CO_2_. In this study, an improved D-lactate production was observed compared to the control strain without PFL and PFL-AE. Especially, during anabolism, positive effects were determined due to the recombinant expression of *C. pasteurianum*’s PFL. During this work, the promoter system, used to control the expression of the recombinant *ldhD* gene, was exchanged to the lactate-inducible P_*lctA*_-promoter originated from *A. woodii*, which also caused an increase in D-lactate production. Cultivation of these P_*lctA*_ promoter strains in a stirred-tank bioreactor (STR) resulted in elevated D-lactate and acetate concentrations compared to experiments in serum bottles and showed increased D-lactate accumulation during expression of the PFL compared to the strain carrying only the *ldhD* gene. A maximum D-lactate concentration of 21.0 ± 2.4 mM was reached in bottle-scale cultivation. Controlled STR batch cultivations with continuous gassing resulted in D-lactate concentrations of up to 34.9 mM. This finding demonstrates that *A. woodii* overexpressing LDHD, PFL, and PFL-AE is a robust strain for the autotrophic production of D-lactate.

**Graphical Abstract:**

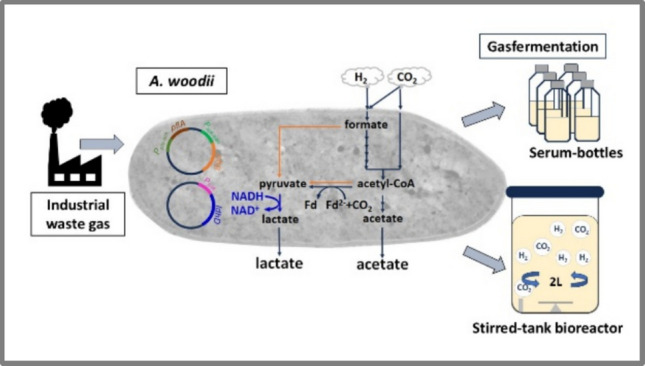

**Supplementary Information:**

The online version contains supplementary material available at 10.1007/s00253-026-13952-5.

## Introduction

Global warming, caused by the greenhouse effect, is one of the main challenges of the twenty-first century. The continuously increasing emissions of the greenhouse gas CO_2_ in our atmosphere are one of the driving forces of global warming (Ripple et al. 2025). Some of these CO_2_ emissions are considered hard to abate, e.g., concrete or steel production. The CO_2_ emissions caused by global concrete production amounted in 2023 to 1.57 Gt (Statista 2024), while CO_2_ emissions caused by global crude steel production amounted to 1.90 Gt in the same year (Statista 2025). One approach to mitigating the greenhouse effect is to capture and reuse CO_2_ from industrial waste gas through gas fermentation. In this process, anaerobic acetogens utilize waste gases containing gaseous C1 carbon sources, such as CO_2_ and CO, into higher-value products (Bengelsdorf and Dürre 2017). *Acetobacterium woodii* is such an anaerobic acetogen and reduces CO_2_ while oxidizing H_2_ via the Wood-Ljungdahl pathway to acetyl-CoA (Balch et al. 1977; Ljungdahl 1986; Drake et al. 2008). During anabolism, acetyl-CoA is primarily converted to pyruvate, since pyruvate is a central metabolite for, e. g., amino acid production or cell wall formation. The pyruvate:ferredoxin oxidoreductase (PFOR) performs the pyruvate synthesis from acetyl-CoA and CO_2_, which requires reduced ferredoxin (Fd^2−^) (Blamey and Adams 1993). The Gibbs free energy change of the PFOR reaction (CO_2_ + acetyl-CoA + Fd^2−^ ⇌ pyruvate + CoA + Fd) is Δ_r_*G*′^m^ = 18.0 ± 13.1 kJ·mol⁻^1^ (calculated with eQuilibrator under the conditions, pH 7.0, reactant concentrations of 1 mM). If cells switch to catabolism, most of the acetyl-CoA is converted to acetate via acetyl-phosphate by the enzymes phosphotransacetylase and acetate kinase. Thereby, the reaction from acetyl-phosphate to acetate gains one ATP per acetate. A lesser portion of acetyl-CoA is then converted to pyruvate via PFOR.

In autotrophically cultivated strains of *A. woodii* P_*bgaL*__LDHD (Mook et al. 2022) and *A. woodii* P_*lctA*__LDHD (Stock et al. 2026), a recombinantly expressed D-lactate dehydrogenase (LDHD) originated from *Leuconostoc mesenteroides* (Li et al. 2012) converted pyruvate to D-lactate. In these recombinant *A. woodii* strains, the genes *lctB*, *lctC*, and *lctD* (Awo_c08710 – Awo_c08730) encoding the LDH/Etf complex are deleted to prevent D-lactate consumption by *A. woodii*. D-lactate is a valuable commodity with a variety of applications in *pharmaceuticals*, cosmetics or, as poly lactic acid, a biodegradable plastic (Narayanan et al. 2004; Song et al. 2012). Lactate is predominantly generated through bacterial fermentation of carbohydrates by lactic acid bacteria and rarely via chemical synthesis (Narayanan et al. 2004). The Gibbs free energy of LDHD’s catabolic reaction, pyruvate + NADH ⇌ lactate + NAD^+^, at pH 7 is Δ_r_*G*′^m^ = −26.8 ± 0.9 kJ mol⁻^1^ (calculated with eQuilibrator). The Gibbs free energy for the overall reaction from acetyl-CoA to D-lactate via PFOR and LDHD is Δ*G*_total_ = − 5.7 ± 13.1 kJ mol⁻^1^. A decrease in pH leads to a further reduction in the Gibbs free energy of this combined reaction (Table 1). Since *A. woodii* produces high titers of acetate during H_2_ and CO_2_ fermentation, the pH decreases continuously without pH control. Thus, the combined reaction of PFOR and LDHD towards D-lactate becomes increasingly favored during batch cultivation.

**Table 1:** Overview of Gibbs free energies of the combined reaction of PFOR and LDHD and PFL and LDHD at pH 7.0, 6.5, and 6.0 (single reactions’ changes in Gibbs free energies calculated with eQuilibrator)

pH [−]	Gibbs free energy [kJ mol^−1^] of the combined reaction of PFOR and LDHD	Gibbs free energy [kJ mol^−1^] of the combined reaction of PFL and LDHD
7.0	− 8.8 ± 13.1	− 7.0 ± 3.0
6.5	− 15.0 ± 13.1	− 10.4 ± 3.0
6.0	− 22.0 ± 13.1	− 14.5 ± 3.0

NADH needed for D-lactate synthesis is supplied by the Rnf complex (Westphal et al. 2018). In *A. woodii* P_*bgaL*__LDHD, the expression of *ldhD* is regulated by the lactose-inducible promoter system P_*bgaL*_ (originated from *Clostridium perfringens*, Hartman et al. 2011; Banerjee et al. 2014), which is a strong promoter in *A. woodii*, resulting in high expression of the *ldhD* gene (Beck et al. 2020)*.* After the induction of *A. woodii* P_*bgaL*__LDHD cells with lactose, growth stopped immediately (Mook et al. 2022), considering a stop of anabolism and the onset of catabolism.

**Fig. 1:**
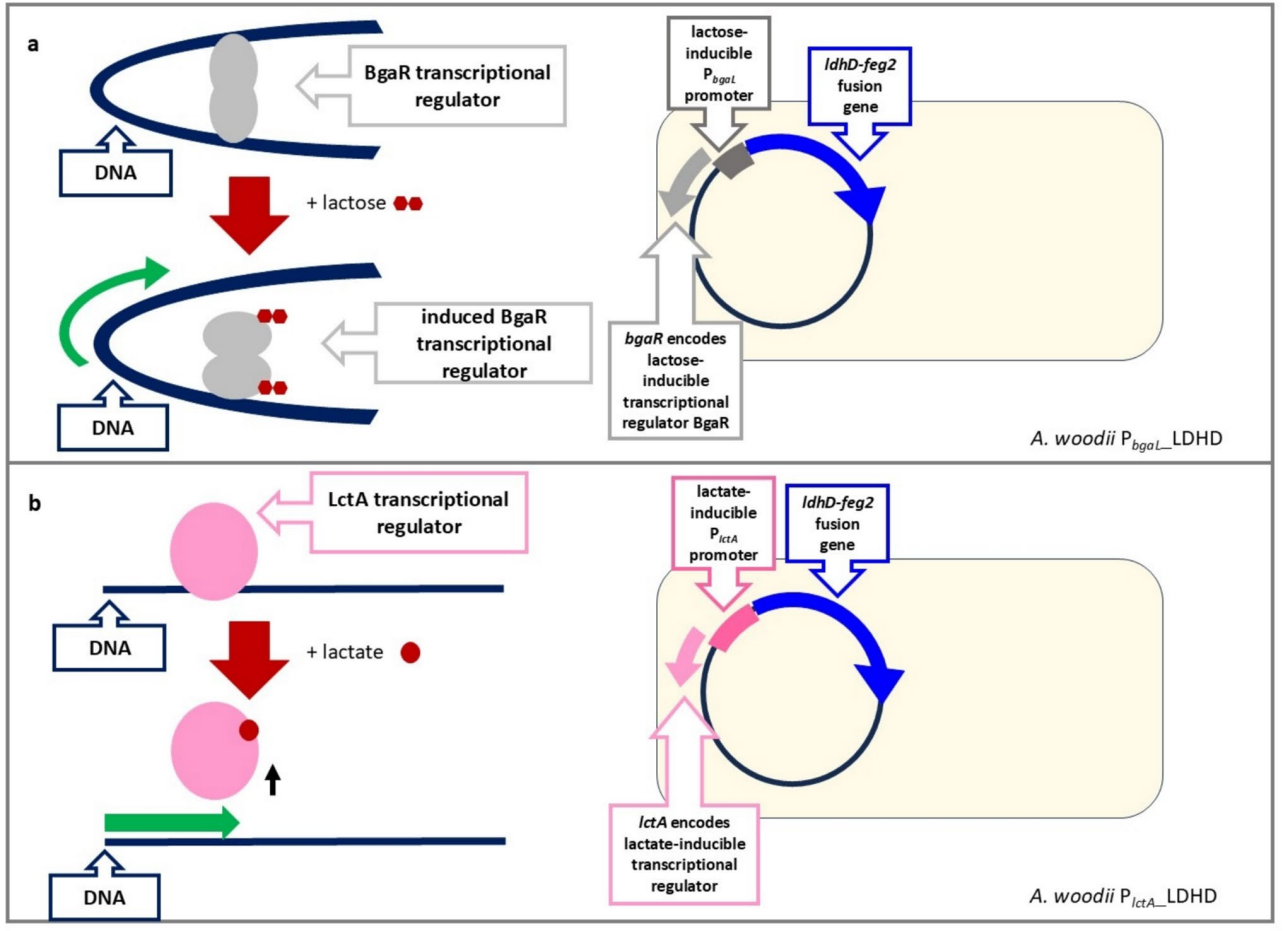
Schematic illustration of *A. woodii ∆pyrE ∆lctBCD* [pMTL83251_P_*bgaL*__NFP], further abbreviated as *A. woodii* P_*bgaL*__LDHD and *A. woodii ∆pyrE ∆lctBCD* [pMTL83251_P_*lctA*__NFP], further abbreviated as *A. woodii* P_*bgaL*__LDHD. **a** Illustration of the transcription regulation of BgaR (bright grey) after induction (red) with lactose (red), transcription direction indicated by a green arrow, and *A. woodii* P_*bgaL*__LDHD, with P_*bgaL*_ (grey) and the gene *bgaR* (bright grey) encoding BgaR regulating the *ldhD-feg2* fusion gene (blue) expression. **b** Illustration of the transcription regulation of LctA (rose) after induction (red) with lactate (red), transcription direction indicated by a green arrow, and *A. woodii* P_*lctA*__LDHD, with P_*lctA*_ (pink) and the gene *lctA* (rose) encoding LctA regulating *ldhD-feg2* fusion gene (blue) expression

In *A. woodii* P_*lctA*__LDHD, the expression of *ldhD* is regulated by the D-lactate-inducible P_*lctA*_ promoter system originated from *A. woodii* itself (Fig. 1). The P_*lctA*_ promoter contains the *lctA* gene, which encodes the repressor LctA and the LctA binding motif. The LctA repressor regulates the *lct* operon natively in *A. woodii* by binding to the DNA upstream of *lctB* (Schoelmerich et al. 2018). LctA is displaced from the DNA in the presence of D-lactate, and the genes of the *lctBCDEF* operon are expressed (Schoelmerich et al. 2018).

To improve D-lactate production by lowering the metabolic burden caused by a plasmid, the integration of the *ldhD* gene into *A. woodii*’s genomic DNA was performed by making use of its endogenous CRISPR/Cas system, which belongs to the Type I-B systems (Poulalier-Delavelle et al. 2023).

**Fig. 2:**
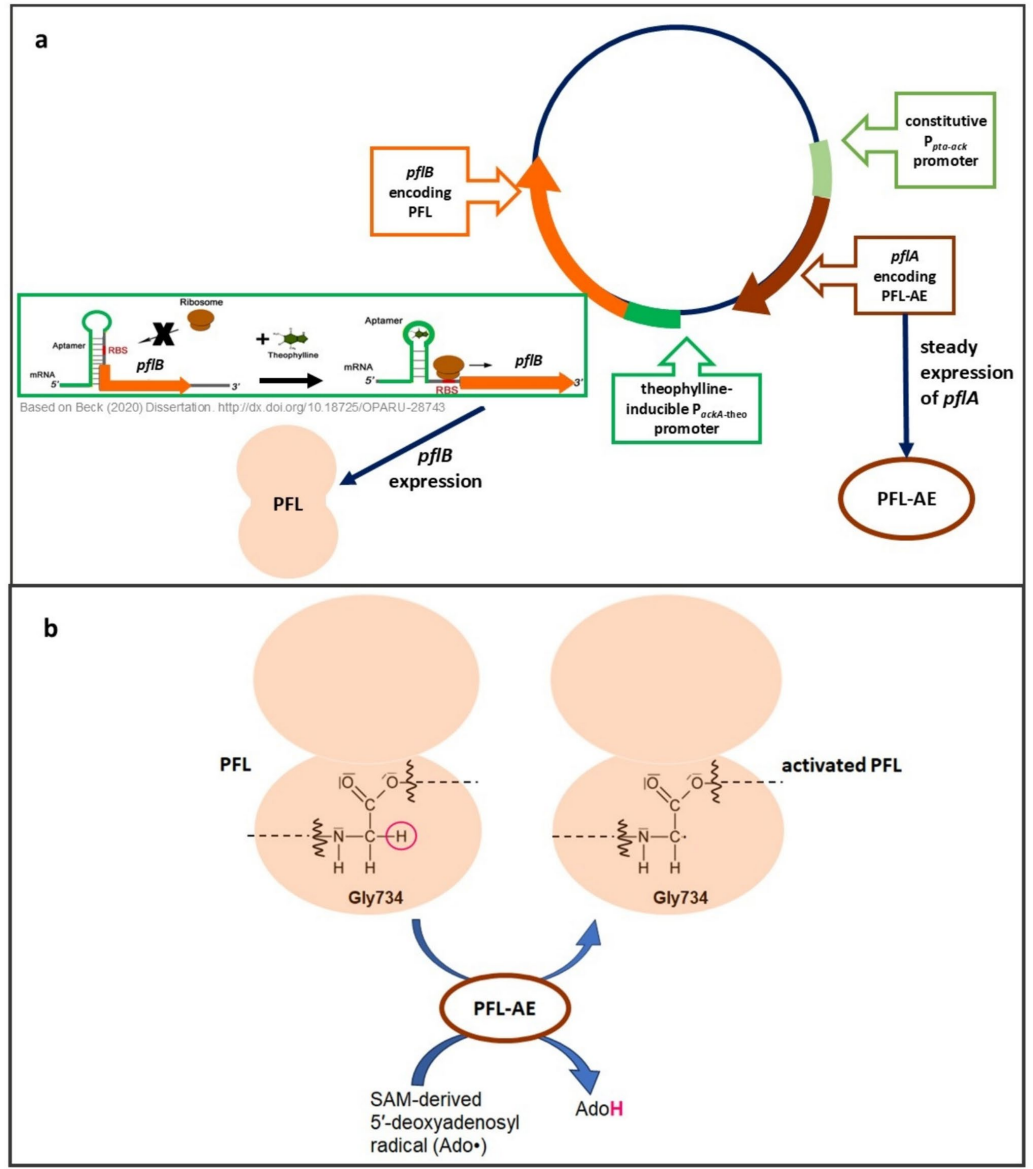
Schematic illustration of the regulation of *pflB* and the activation of the PFL by PFL-AE. **a** pMTL871ksb_P_*pta-ack*__*pflA*_P_*ackA*-theo__*pflB* with P_*pta-ack*_ (pastel green) regulating the expression of *pflA* (brown) constitutively and P_*ackA.*theo_ (forest green), which is inducible by theophylline, regulating the expression of *pflB* (orange). **b** Activation of the PFL by its activating enzyme, generating a glycyl radical at Gly734, while a *S*-adenosyl-l-methionine (SAM) derived 5′-deoxyadenosyl radical (Ado·) is converted to 5′-deoxyadenosine (AdoH, based on Moody et al. 2023)

**Fig. 3:**
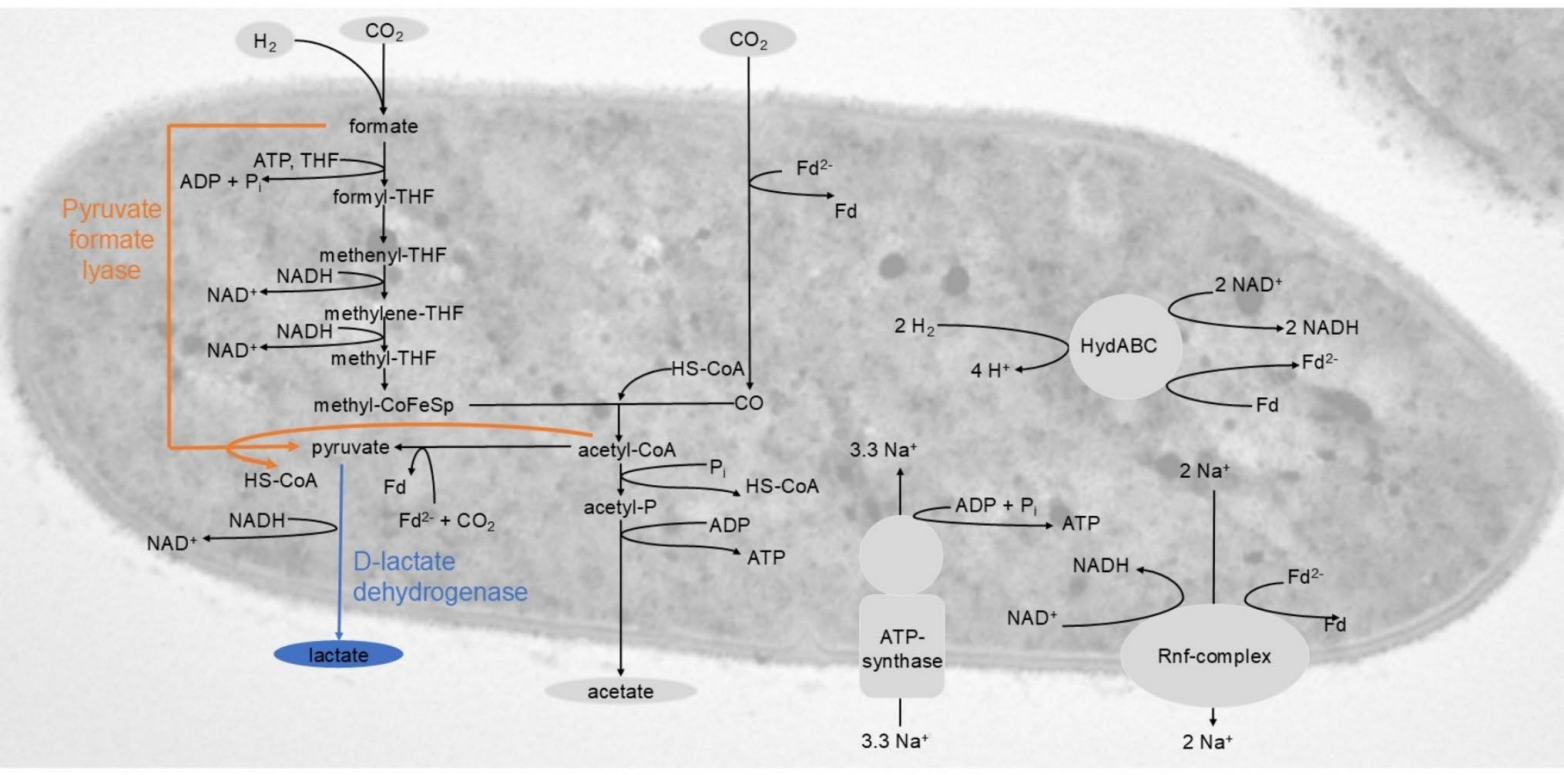
Illustration of the planned metabolic engineering based on the parental strain *A. woodii ∆pyrE ∆lctBCD*. WLP and energy conservation-associated enzymes (Rnf-complex, HydABC, ATPase) are marked in grey. LDHD reaction marked in blue. PFL branching the methyl branch of the WLP is marked in orange

A further strategy to enhance D-lactate production aimed to express an additional recombinant enzyme to branch the methyl branch of the WLP to increase the intracellular pyruvate level from formate and acetyl-CoA (Fig. 3). Therefore, the genes encoding the pyruvate formate lyase (PFL) of *C. pasteurianum* and its activating enzyme (PFL-AE, Weidner and Sawers 1996) are recombinantly expressed in *A. woodii* (Fig. 2a). The PFL is a glycyl radical enzyme and requires a pyruvate formate lyase activating enzyme (PFL-AE). The activation of PFL happens when the PFL-AE forms a specific glycyl radical in the PFL (Crain and Broderick 2013, Fig. 2b).

The PFL usually works under anaerobic conditions and catalyzes the reaction from pyruvate and CoA to formate and acetyl-CoA. Hong et al. (2020) observed a reverse reaction of the native PFL towards pyruvate in *C. pasteurianum* if there is a formate excess (Hong et al. 2020). Im et al. (2025) expressed a PFL originated from *A. woodii* and its activating enzyme in *C. ljungdahlii* and cultivated the recombinant strains in the presence of formate excess. Increased growth, along with enhanced product concentration, was observed, indicating a benefit of expressing the PFL in relation to formate utilization and pyruvate availability (Im et al. 2025). Under autotrophic conditions, *A. woodii* produces formate due to the hydrogen-dependent CO_2_ reductase (= HDCR, Schuchmann and Müller 2013), and sufficient ATP availability enables the conversion from formate to formyl-THF with the formyl-THF synthetase. Under a formate excess, the recombinantly established PFL of *C. pasteurianum* in *A. woodii* could catalyze, although thermodynamically unfavorable, the reaction of formate and acetyl-CoA to pyruvate and coenzyme A (Fig. 3). This would fill up pyruvate reservoirs by branching the methyl branch of the Wood-Ljungdahl pathway. The following three considerations support this hypothesis. The known *K*_m_ values for the reaction from formate and acetyl-CoA to pyruvate and CoA in *E. coli* via PFL are 24.5 mM for formate and 0.051 mM for acetyl-CoA (Knappe et al. 1974) and are presumably equal in *A. woodii*. At a pH of 7.0, the Gibbs free energy change of the PFL reaction formate + acetyl-CoA ⇌ pyruvate + CoA is Δ_r_*G*′^m^ = 19.8 ± 2.9 kJ mol^−1^ (calculated with eQuilibrator), which is in a similar range to the Gibbs energy of the PFOR reactions towards pyruvate. Moreover, the total Gibbs energy of the combined reaction of PFL and LDHD corresponds to the Gibbs energy of the combined reaction of PFOR and LDHD at the pH values between 7.0 and 6.0 (Table 1). Last, the PFOR requires Fd^2−^ to convert acetyl-CoA and CO_2_ to pyruvate, while the PFL only requires acetyl-CoA and formate, but no Fd^2−^ to generate pyruvate.

Therefore, in this study, we aimed to enhance autotrophic D-lactate production in *A. woodii* by combining promoter engineering and metabolic pathway extension. We replaced the growth-inhibiting P_*bgaL*_ promoter with the native, lactate-induced P_*lctA*_ promoter to enable sustained LDHD expression. Additionally, we introduced the heterologous PFL/PFL-AE system to increase intracellular pyruvate availability and therefore lactate yields. We compared autotrophic lactate production of the resulting strains in serum bottle experiments and, subsequently, in continuously gassed stirred tank reactor (STR) batch cultivations.

## Materials and methods

### Medium and cultivation

The *Escherichia coli* XL1-Blue cells (Agilent, USA) used for plasmid construction were cultivated in Luria–Bertani (LB) medium, consisting of tryptone (10 g/L), NaCl (10 g/L), and yeast extract (5 g/L) (Bertani 1951). The cells were cultivated in liquid LB medium while shaking (180 rpm) or on agar plates (1.5% (w/w) agar) at 37 °C. The LB medium was supplemented with 30 µg/mL chloramphenicol.

All *A. woodii* strains were cultivated in modified DSM 135 medium (Hoffmeister et al. 2016). The medium was supplemented with uracil (20 mg/L), to grow the *A. woodii* strains harboring a *ΔpyrE* deletion (uracil auxotrophy, Baker et al. 2022) at 30 °C. The gas atmosphere in the serum bottles (SGD pharma, Paris, France) was changed seven times with 80% N_2_ and 20% CO_2_ before autoclaving to ensure that the medium is anaerobic. *A. woodii* P_*bgaL*__LDHD and *A. woodii* P_*lctA*__LDHD cells were cultivated in the presence of 5 µg/mL clarithromycin, and *A. woodii* P_*bgaL*_*_*LDHD_P_*ackA-*theo__PFL and *A. woodii* P_*lctA*_*_*LDHD_P_*ackA-*theo__PFL cells were supplemented with 5 µg/mL clarithromycin and 15 µg/mL thiamphenicol. All *A. woodii* strains were first grown heterotrophically in 5 mL medium containing 40 mM fructose as the carbon source to obtain cell mass for subsequent autotrophic experiments. The respective pre-cultures and main-cultures of all *A. woodii* strains were cultivated autotrophically in 500-mL serum bottles (SGD Pharma, Paris, France) containing 50 mL medium and 33% CO_2_ and 67% H_2_, serving as carbon and energy source. In the autotrophic growth experiments, a headspace pressure of 1000 hPa was used.

In the continuously gassed batch experiments, strains were cultivated autotrophically in a 3.6 L STR (KLF, Bioengineering, Wald, Switzerland) with a working volume of 2.0 L. The bioreactor was gassed at a flow rate of 0.084 vvm using a sintered sparger located at the bottom of the reactor. Independent mass flow controllers (F-201CV-500 RGD-33-V, Bronkhorst High-Tech BV, Ruurlo, Netherlands) enabled the precise addition of specific gas components at desired ratios. During all STR cultivations, the inlet gas phase consisted of 33% (v/v) CO_2_ and 67% (v/v). Prior to cultivation, the bioreactor was sterilized at 121 °C for 20 min, already containing all relevant media components (except for L-cysteine HCl, MgSO_4_, trace elements, and vitamins). Starting during cool down, the bioreactor was flushed with the respective gas mixture for at least 12 h to provide anaerobic conditions. Four hours prior to inoculation, the remaining media components were added through sterile filters (pore size, 0.2 µm, VWR, Radnor, PA, USA). Batch cultivations were conducted without the addition of antibiotics. The temperature was set to 30 °C, and the pH was controlled at pH 7.0 ± 0.05 using 5 M KOH or 95% (w/w) H_2_SO_4_. Prior to inoculation, cells were anaerobically transferred to several 50-mL tubes (Greiner centrifuge tubes T2193, Merck KGaA, Darmstadt, Germany) and centrifuged (20 min, 3620 g (rcf), Rotica 50 RS, Hettich GmbH & Co. KG, Tuttlingen, Germany). Subsequently, the supernatant was discarded, and the remaining pellets were resuspended in anaerobic PBS buffer. The STR was inoculated at an initial OD_600_ of 0.1.

The *A. woodii* P_*bgaL*__LDHD cells in all main-cultures were induced with 2.5 mM lactose, the *A. woodii* P_*bgaL*_*_*LDHD_P_*ackA-*theo__PFL cells with 2.5 mM lactose and 1.0 mM theophylline, and the *A. woodii* P_*lctA*_*_*LDHD_P_*ackA-*theo__PFL cells with 1.0 mM theophylline at an OD_600_ of 0.6 or 0.7, respectively.

### Plasmid and strain construction

The plasmid pMTL-MPD21 (Poulalier-Delavelle et al. 2023) was used to construct the *pheA*-knock-out strain *A. woodii ∆pyrE ∆lctBCD ∆pheA.* The primers ColE1 + tra-F2 and pCD6-R1 were used to verify the presence of the plasmid in electroporated *A. woodii ∆pyrE ∆lctBCD* cells. The primers FW_pheAHA1.5_out and RV_pheAHA1.5_out were used to verify the *pheA* deletion in the genome of *A. woodii ∆pyrE ∆lctBCD* caused by the plasmid pMTL-MPD21 (Table S1). After pMTL-MPD21 was cured. The plasmid pMTL-MPD23_P_*bgaL*__ldhD was constructed by linearizing the *pheA*-reconstruction plasmid pMTL-MPD23 (Poulalier-Delavelle et al. 2023) with the enzymes *Xba*I and *Not*I. The lactose-inducible P_*bgaL*_ promoter fragment was amplified from the template pMTL83251_P_*bgaL*__NFP using the CloneAmp™ HiFi PCR premix (Takara Bio Europe) and the primers P_*bgaL*__fwd and P_*bgaL*__rev. The gene *ldhD* was amplified from the plasmid pJIR750_P_tet__ldhD_LM (Beck 2020) using the CloneAmp™ HiFi PCR premix (Takara Bio Europe) and the primers ldhD(LM)_fwd and ldhD(LM)_rev (Table S1). The plasmid pMTL-MPD23_P_*bgaL*__LDHD was assembled using the NEBuilder® HiFi DNA Assembly Kit (New England Biolabs, Ipswich, Ma, USA). After replication in chemocompetent *E. coli* XL1-Blue cells (Green and Sambrook 2012, Agilent, USA), the plasmid DNA was isolated (Zyppy™ Plasmid Miniprep Kit, Zymo Research, USA) and transformed into the strain *A. woodii ∆pyrE ∆lctBCD ∆pheA* following the protocol from Baur et al. (2025) to construct the strain. The plasmid pMTL-MPD23_P_*bgaL*__ldhD carried two homology arms. The upstream arm carried the functional *pheA* gene to restore *pheA* in the genome using *A. woodii*’s endogenous CRISPR/Cas system and homologous recombination (Poulalier-Delavelle et al. 2023) *A. woodii pheA*_+_*::ldhD*. Since this strain produced less D-lactate than the *A. woodii* strain harboring the plasmid pMTL83251_P_*bgaL*__NFP (Mook et al. 2022), a plasmid-based approach was pursued.

**Fig. 4:**
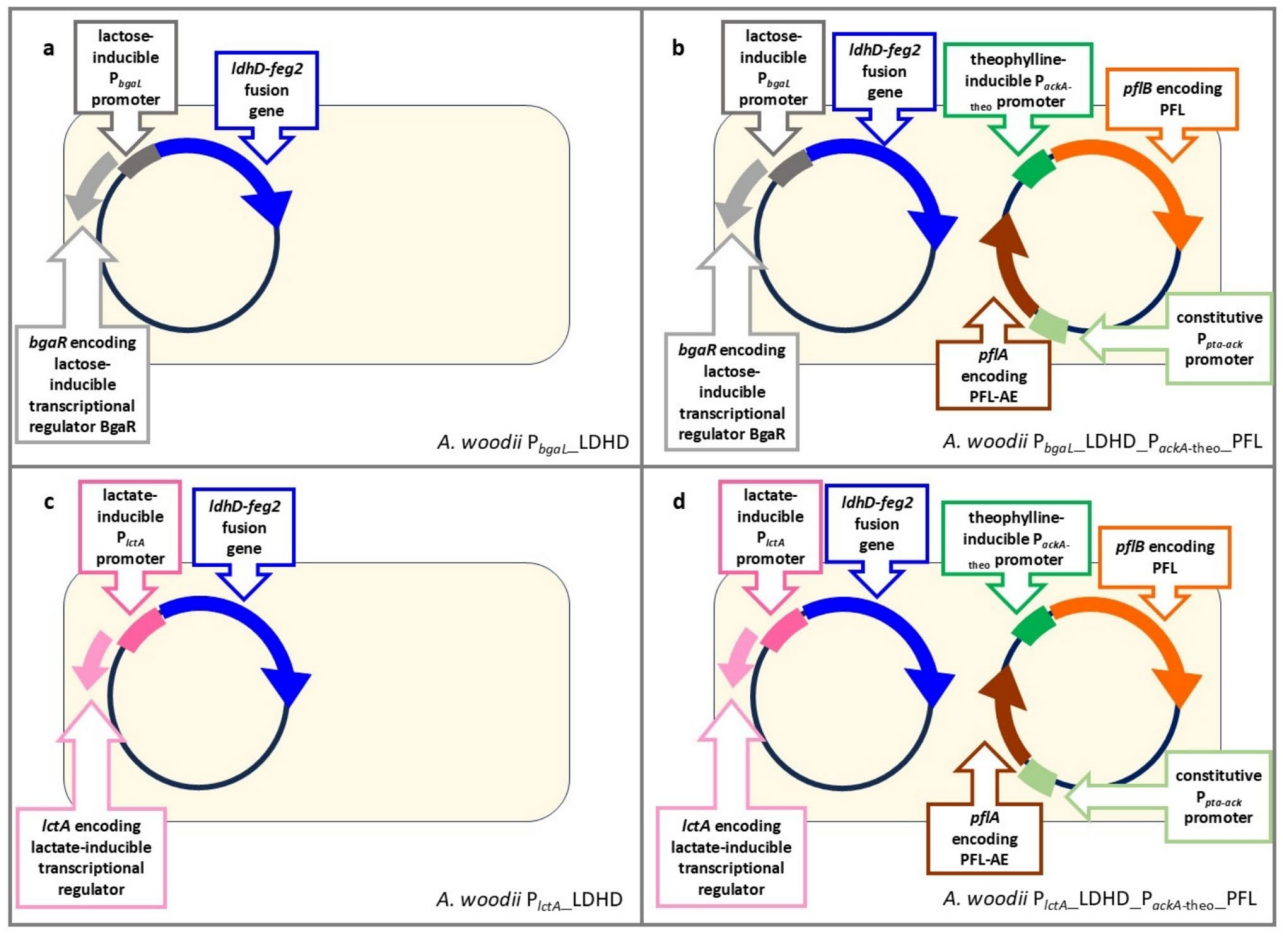
Schematic illustration of constructed *A. woodii* strains. **a ***A. woodii ∆pyrE ∆lctBCD* [pMTL83251_P_*bgaL*__NFP] further abbreviated as *A. woodii* P_*bgaL*__LDHD, with P_*bgaL*_ (grey, see Fig. 1) regulating NFP (blue) expression. **b ***A. woodii ∆pyrE ∆lctBCD* [pMTL83251_P_*bgaL*__NFP] [pMTL871ksb_P_*pta-ack*__*pflA*_P_*ackA*-theo__*pflB*] further abbreviated as *A. woodii* P_*bgaL*__LDHD_P_*ackA-*theo__PFL with a two-plasmid system harboring pMTL83251_P_*bgaL*__NFP with P_*bgaL*_ (grey) regulating the expression of the gene encoding NFP (blue) and pMTL871ksb_P_*pta-ack*__*pflA*_P_*ackA*-theo__*pflB* with P_*pta-ack*_ (pastel green) regulating the expression of *pflA* (brown) and P_*ackA.*theo_ (forest green, see Fig. 2) regulating the expression of *pflB* (orange). **c*** A. woodii ∆pyrE ∆lctBCD* [pMTL83251_P_*lctA*__NFP] further abbreviated as *A. woodii* P_*lctA*__LDHD, with P_*lctA*_ (pink, see Fig. 1) regulating NFP (blue) expression. **d*** A. woodii ∆pyrE ∆lctBCD* [pMTL83251_P_*lctA*__NFP] [pMTL871ksb_P_*pta-ack*__*pflA*_P_*ackA*-theo__*pflB*] further abbreviated as *A. woodii* P_*lctA*__LDHD_P_*ackA-*theo__PFL, with a two-plasmid system harboring pMTL83251_P_*lctA*__NFP with P_*lctA*_ (pink) regulating the expression of the gene encoding NFP (blue) and pMTL871ksb_P_*pta-ack*__*pflA*_P_*ackA*-theo__*pflB* with P_*pta-ack*_ (pastel green) regulating the expression of *pflA* (brown) and P_*ackA.*theo_ (forest green, see Fig. 2) regulating the expression of *pflB* (orange)

The plasmid pMTL83251_P_*bgaL*__NFP (Fig. 4a) contains the *ldhD-feg2* fusion gene (GenBank-Nr. OL439953, Mook et al. 2022) under the control of the lactose-inducible promoter system P_*bgaL*_ originated from *Clostridium perfringens* (Hartman et al. 2011; Mook et al. 2022). The plasmid pMTL83251_P_*lctA*__NFP (Fig. 4c) contains the *ldhD-feg2* fusion gene (GenBank-Nr. OL439953, Mook et al. 2022), which is controlled by the lactate-inducible promoter system P_*lctA*_ originated from *A. woodii* itself (Stock et al. 2026).

The plasmid pMTL871ksb_P_*pta-ack*__*pflA*_P_*ackA*-theo__*pflB* was constructed to establish a recombinantly produced pyruvate-formate lyase (PFL) and its activating enzyme (PFL-AE) in *A. woodii* P_*bgaL*__LDHD and *A. woodii* P_*lctA*__LDHD (Table 3). This new plasmid was constructed using the linearized (restriction enzyme *Apa*I) *Clostridium* shuttle plasmid DNA of pMTL87151. Its constitutive promoter, P_*pta-ack*_, originates from *Clostridium ljungdahlii* and was amplified using the primers P_*pta-ack*__fwd and P_*pta-ack*__rev with the respective genomic DNA as a template. The genomic DNA was isolated using the MasterPure™ Gram-Positive DNA Purification Kit (Biosearch, LGC, UK). The gene *pflA* (AQ983_RS00460) encoding the pyruvate formate lyase activating enzyme, originated from *C. pasteurianum*, was amplified using the primers pflA_fwd and pflA_rev (Table S1). The plasmid pMTL871ksb_P_*pta-ack*__pflA was assembled using the NEBuilder® HiFi DNA Assembly Kit (New England Biolabs, Ipswich, Ma, USA) and replicated in chemocompetent *E. coli* XL1-Blue cells (Green and Sambrook 2012, Agilent, USA). This plasmid was again isolated from *E. coli* XL1-Blue and linearized using the restriction enzymes *Xho*I and *Bam*HI. The gene *pflB* (AQ983_RS00455) encoding the pyruvate formate lyase, also originated from *C. pasteurianum*, was amplified using the primers pflB_fwd and pflB_rev (Table S1). The theophylline-inducible P_*ackA*-theo_ promoter (Beck 2020) was chosen to control the expression of this *pflB* gene and was amplified using the primers P_*ackA*-theo__fwd and P_*ackA*-theo__rev (Table S1). The plasmid pMTL871ksb_P_*pta-ack*__pflA_P_*ackA*-theo__pflB (Fig. 4b, d) was assembled using the NEBuilder® HiFi DNA Assembly Kit (New England Biolabs, Ipswich, Ma, USA) and replicated in chemocompetent *E. coli* XL1-Blue cells (Green and Sambrook 2012, Agilent, USA). All plasmids were verified either by whole-plasmid sequencing (Plasmid-EZ, GENEWIZ Germany GmbH) or by Sanger sequencing (GENEWIZ Germany GmbH or Microsynth AG, Switzerland).

All constructed plasmids were transformed into the respective *A. woodii* strains through electroporation following the protocol described by Baur et al. (2025). All strains were verified by means of enzymatic digestion of the respective plasmid or direct PCR tests. Corresponding methods and results are published elsewhere (Baur et al. 2025).

### Analytics

At each time point during the serum bottle growth experiments, 2 mL of cell suspension was withdrawn to monitor OD_600_, pH-values of the respective cell suspension, and metabolic products. The headspace pressure in the serum bottles was measured by using a handheld manometer (Wika, Germany) before sampling. The total pressure loss in the serum bottles over time reflects the consumption of the gas containing 67% H_2_ and 33% CO_2_ (MTI IndustrieGase AG, Neu-Ulm, Germany) during the autotrophic growth experiments. The OD_600_ was determined using a UV/Visible spectrophotometer (Ultrospec 3100 pro, Amersham Biosciences, UK). The metabolic products were determined in the supernatant of the cell suspensions. Therefore, these were centrifuged at 13,000 × g at 4 °C for 30 min. Five hundred microliters of supernatant was aliquoted into vials (Bottle R1, CS Chromatographie, Germany) and measured via high-performance liquid chromatography (HPLC) using the Agilent 1260 Infinity II HPLC system (Agilent Technologies, Santa Clara, CA, USA). The lactate, formate, and acetate concentrations were detected using a diode array detector. A 300 × 8 mm column packed with polystyrene-divinylbenzene (PS-DVB) copolymer (CS - Chromatographie Service GmbH, Germany) and heated to 60 °C was used for the separation of 20 µL supernatant per injection. As a mobile phase, 5 mM H_2_SO_4_ was used with a flow rate of 0.8 mL min^− 1^. The data analysis was performed using the OpenLab CDS ChemStation Edition A.01.03 software package (Agilent Technologies, Santa Clara, CA, USA).

During STR cultivations, at each time point, up to 5 mL sample was withdrawn through a septum (*d* = 12 mm, Infors AG, Bottmingen, Germany) to determine optical density in technical triplicates with an UV/Visible spectrophotometer (Genesys 10S UV-Vis, Thermo Scientific, Neuss, Germany) and product concentrations via HPLC (LC-2030C, Shimadzu, Kyoto, Japan) analysis. Prior to product determination, samples were filtered (Chromafil RC20/15 MS; Macherey-Nagel GmbH & Co.KG, Düren, Germany) and subsequently stored at 4 °C. Product concentrations were determined using a cation exchange column (Aminex HPX-87H, Bio-Rad, Munich, Germany) and a refractive index detector (RID-20A, Shimadzu, Kyoto, Japan). Measurements were conducted with 10 µL sample at a solvent (5 mM H_2_SO_4_) flow rate of 0.6 mL min^−1^ and a column temperature of 60 °C. The pH and the redox potential were continuously measured using autoclavable sensors (405-DPAS-SC-K8s/120, Mettler Toledo, Giesen, Germany, and Pt4805-DPAS-SC-K8S/120, Mettler Toledo, Germany, respectively). The volumetric exhaust gas flow rate was monitored in a 20 s interval using a mass flow meter (MFM, F-111B-1K0-RGD-33-E; Bronkhorst High-Tech B.V., Ruurlo, Netherlands). H_2_ and CO_2_ uptake rates (H_2_UR and CO_2_UR) were calculated using data from the thermal MFM that measured input and exhaust gas flow rates (F-111B-1K0-RGD-33-E; Bronkhorst High-Tech B.V., the Netherlands) and a micro gas chromatograph (µGC, micro-GC 450, Agilent Technologies, Waldbronn, Germany) that measured gas compositions in the exhaust gas in a 10 min interval. The µGC contained a 1 m Cox HI column (molecular sieve, nitrogen as carrier gas, column temperature 100 °C, initial pressure 200 kPa) and a thermal conductivity detector (Agilent Technologies, Waldbronn, Germany).

Individual H_2_ and CO_2_ uptake rates were calculated from changes in the component-specific molar exhaust gas flows. Since MFM measurements depend on the exact gas mixture, the measured volumetric exhaust gas flow was corrected using a mixed-gas conversion factor, calculated from the gas conversion factors (C) of each individual gas component (*G*_*i*_) and their respective molar fraction at this time point (*y*_*i*_). Each single gas conversion factor was determined empirically from the slopes of respective calibration curves, where an increase in pure gas flow was correlated to the increase measured by the MFM. The mixed-gas conversion factor was calculated for every time point during cultivation according to the following equation:

1 $$C= \frac{100}{{\sum}_{i=1}^{n}\frac{{y}_{i}}{{G}_{i}}}$$

All measured exhaust gas flow rates were corrected for gas composition using the term *C*. The molar exhaust gas flow of each component was then calculated from the corrected total exhaust gas flow rate and the corresponding exhaust gas fraction determined by µGC according to the following equation:

2 $${\dot{n}}_{i,out}\left(t\right)=\frac{{\dot{V}}_{gas,out}\left(t\right)\bullet {\dot{y}}_{i,out}\left(t\right)}{{V}_{m}}$$

where ${\dot{n}}_{i,out}\left(t\right)$ is the molar exhaust gas flow of component *i*, ${\dot{V}}_{gas,out}\left(t\right)$ is the exhaust gas flow rate, ${\dot{y}}_{i,out}\left(t\right)$ is the molar fraction of component *i* in the exhaust gas, and ${V}_{m}$ is the molar gas volume. The molar exhaust gas flow at the beginning of the cultivation, when no biological gas conversion was observed, was used as the reference value. Thus, the uptake rate of each gas component *i* was calculated as the difference between the molar exhaust gas flow rates of each component at each time point and the respective flow rate in the beginning.

### Statistics

During serum bottle cultivations, a one-sided *t*-test was performed to determine if the different *A. woodii* strains showed a significantly higher maximum lactate concentration, maximum lactate:acetate ratio, maximum specific lactate concentration, or maximum lactate production rate than the others. To determine the *p*-value, Welch’s degrees of freedom were incorporated. *p*-values lower than 0.05 indicate that one determined value is significantly higher.

## Results

### Strain construction

*A. woodii ∆pyrE ∆lctBCD* served as a parental strain for all constructed and verified recombinant *A. woodii* strains (Table 2). *A. woodii ΔpyrE ΔlctBCD ΔpheA* additionally displays a phenylalanine auxotrophy caused by the deletion of the *pheA* gene encoding the prephenate dehydratase (Figure S1). Since all strains harbor the *∆pyrE* and *∆lctBCD* deletions, this genetic background is not further mentioned in the text to simplify strain labelling. The *pheA* gene was reconstructed via homologous recombination in *A. woodii pheA*_+_*::ldhD*. The *ldhD* gene, which originated from *Leuconostoc mesenteroides*, was regulated by the lactose-inducible P_*bgaL*_ promoter system and genome-integrated downstream of *pheA* (Figure S2). In *A. woodii* P_*bgaL*__LDHD and *A. woodii* P_*lctA*__LDHD, the plasmid-borne production of a lactate dehydrogenase (encoded by *ldhD*, codon-optimized for *A. woodii*) was regulated by the lactose-inducible P_*bgaL*_ promoter system (Mook et al. 2022) and the lactate-inducible P_*lctA*_ promoter system (Stock et al. 2026), respectively. *A. woodii* P_*bgaL*__LDHD_PFL and *A. woodii* P_*lctA*__LDHD_PFL additionally carry a plasmid containing the *pflB* gene encoding the PFL from *C. pasteurianum* regulated by the theophylline-inducible P_*ackA*-theo_ promoter system and the *pflA* gene encoding its activating enzyme regulated by the constitutive P_*pta-ack*_ promoter system (Table 2). The verification of the *A. woodii* strains via PCR or restriction digest was already published by Baur et al. (2025).

**Table 2:** *A. woodii* strains used in this study

Strain	Genotype	Description	Source
*A. woodii* WT	*Acetobacterium woodii* DSM 1030	*A. woodii* wildtype	DSMZ
*A. woodii ΔpyrE ΔlctBCD*	*A. woodii ∆pyrE ∆lctBCD*	*A. woodii* strain displaying an uracil-auxotrophy and a knock-out of the genes encoding the lactate dehydrogenase, the electron transfer flavoprotein subunit al *pheA* and the electron transfer flavoprotein subunit beta (EtfA and EtfB)	Beck et al. (2020)
*A. woodii ΔlctBCD ΔpheA*	*A. woodii ΔpyrE ΔlctBCD ΔpheA*	Same genotype as *A. woodii ∆pyrE ∆lctBCD* displaying an additional phenylalanine auxotrophy	Baur et al. (2025)
*A. woodii pheA*_+_*::ldhD*	*A. woodii ΔpyrE ΔlctBCD pheA*_+_*::ldhD*	Same genotype as *A. woodii ΔpyrE ΔlctBCD ΔpheA* expressing a genome-integrated *ldhD* gene, downstream of the restored *pheA* gene, originated from *L. mesenteroides* under the control of the lactose-inducible P_*bgaL*_ promoter	Baur et al. (2025)
*A. woodii* P_*bgaL*__LDHD	*A. woodii ∆pyrE ∆lctBCD* [pMTL83251_P_*bgaL*__NFP]	Same genotype as *A. woodii ∆pyrE ∆lctBCD* expressing recombinantly a codon-optimized *feg2-ldhD* fusion gene (N-terminally FAST-tagged LDHD fusion protein (NFP)) under the control of the lactose-inducible P_*bgaL*_ promoter	Mook et al. (2022)
*A. woodii* P_*lctA*__LDHD	*A. woodii ∆pyrE ∆lctBCD* [pMTL83251_P_*lctA*__NFP]	Same genotype as *A. woodii ∆pyrE ∆lctBCD* expressing recombinantly a codon-optimized *feg2-ldhD* fusion gene (N-terminally FAST-tagged LDHD fusion protein (NFP)) under the control of the lactate-inducible P_*lctA*_ promoter	Stock et al. (2026)
*A. woodii* P_*bgaL*__LDHD_P_*ackA-*theo__PFL	*A. woodii ∆pyrE ∆lctBCD* [pMTL83251_P_*bgaL*__NFP] [pMTL871ksb_P_*pta-ack*__*pflA*_P_*ackA*-theo__*pflB*]	Same genotype as *A. woodii ∆pyrE ∆lctBCD* [pMTL83251_P_*bgaL*__NFP] expressing recombinantly *pflA* from *C. pasteurianum* under the control of the constitutive P_*pta-ack*_ promoter, and *pflB* from *C. pasteurianum* under the control of the theophylline-inducible promoter P_*ackA*-theo_	Baur et al. (2025)
*A. woodii* P_*lctA*__LDHD_P_*ackA-*theo__PFL	*A. woodii ∆pyrE ∆lctBCD* [pMTL83251_P_*lctA*__NFP] [pMTL871ksb_P_*pta-ack*__*pflA*_P_*ackA*-theo__*pflB*]	Same genotype as *A. woodii ∆pyrE ∆lctBCD* [pMTL83251_P_*lctA*__NFP] expressing recombinantly *pflA* from *C. pasteurianum* under the control of the constitutive P_*pta-ack*_ promoter, and *pflB* from *C. pasteurianum* under the control of the theophylline-inducible promoter P_*ackA*-theo_	Baur et al. (2025)

### Characterization of the genome integration of *ldhD* in* A. woodii*

Growth and product formation of the genome-integration strain *A. woodii pheA*_+_*::ldhD* was studied in comparison to *A. woodii* P_*bgaL*__LDHD and *A. woodii* WT in autotrophic serum bottle experiments (Figure S4). *A. woodii pheA*_+_*::ldhD* yielded a much lower concentration of D-lactate compared with *A. woodii* P_*bgaL*__LDHD, harboring the plasmid-based system already used by Mook et al. (2022).

*A. woodii pheA*_+_*::ldhD* produced a maximum concentration of 2.6 ± 0.2 mM D-lactate (after induction, Figure S4). This corresponds to a post-induction D-lactate concentration of 2.0 mM, normalized to an OD_600_ of 1 (Table S2), while *A. woodii* P_*bgaL*__LDHD (induced) produced a maximum of 9.1 ± 0.4 mM D-lactate; thereof, 8.6 mM was produced after induction. Normalized to an OD_600_ of 1, this corresponds to a maximum post-induction D-lactate concentration of 11.4 mM OD_600_^−1^. Non-induced *A. woodii pheA*_+_*::ldhD* and *A. woodii* WT cells produced no D-lactate (Table S2).

*A. woodii pheA*_+_*::ldhD* (not induced) reached a maximum OD_600_ of 1.58 ± 0.12, while *A. woodii pheA*_+_*::ldhD* (induced) grew to a peak OD_600_ of 1.33 ± 0.14 (Figure S4 a). The pH value was manually kept between pH 6 and pH 7.5 (Figure S4 b). Acetate was the main product of *A. woodii pheA*_+_*::ldhD* (induced) (326.1 ± 17.2 mM, Figure S4 f) with a maximum post-induction molar lactate:acetate ratio of 0.01 ± 0.0 (Table S2).

The lower lactate production of *A. woodii pheA*_+_*::ldhD* (induced) compared to *A. woodii* P_*bgaL*__LDHD (induced) was the reason to continue with a plasmid-based LDHD approach in the following experiments.

### D-Lactate and formate production achieved by establishing a pyruvate formate lyase (PFL)

**Fig. 5:**
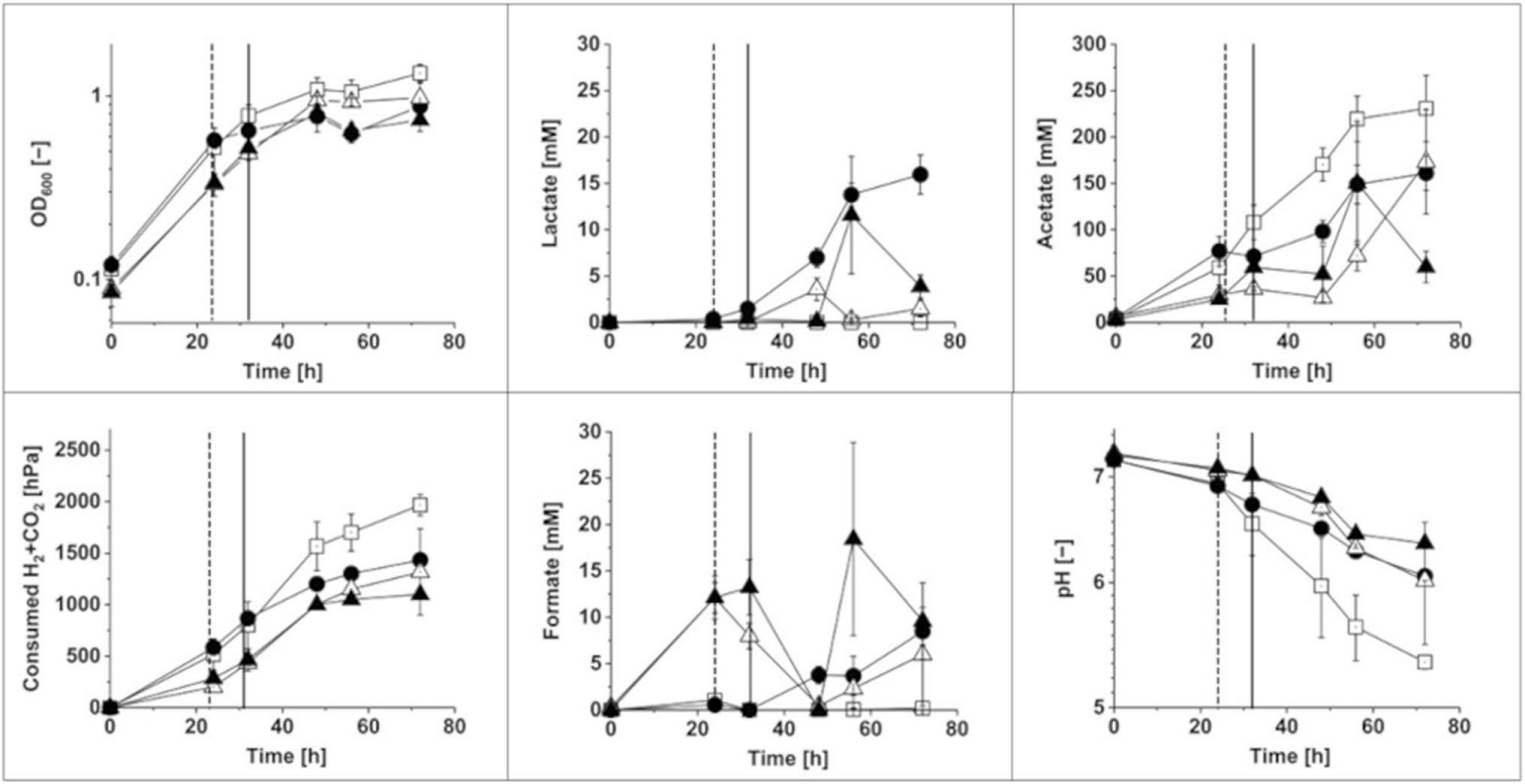
Autotrophic growth experiments in serum bottles with the strains *A. woodii* WT (white squares), *A. woodii* P_*bgaL*__LDHD (lactose-induced, black circles), *A. woodii* P_*bgaL*__LDHD_P_*ackA-*theo__PFL (not induced, white triangles), and *A. woodii* P_*bgaL*__LDHD_P_*ackA-*theo__PFL (lactose- and theophylline-induced, black triangles). OD_600_ (**a**), pH (**b**), acetate concentration (**c**), consumption of H_2_ and CO_2_ (**d**), formate concentration (**e**), and D-lactate concentration (**f**) were monitored. The dotted line shows the induction of the *A. woodii* P_*bgaL*__LDHD cells with 2.5 mM lactose. The full line shows the induction of the *A. woodii* P_*bgaL*__LDHD_P_*ackA-*theo__PFL cells with 2.5 mM lactose and 1 mM theophylline. *n* = 3. Error bars indicate standard deviations

The autotrophic performance of *A. woodii* P_*bgaL*__LDHD_P_*ackA-*theo__PFL was characterized in comparison to *A. woodii* P_*bgaL*__LDHD and *A. woodii* WT (Fig. 5a–f) in serum bottle experiments. *A. woodii* P_*bgaL*__LDHD_P_*ackA-*theo__PFL produced from H_2_ + CO_2_ similar amounts of D-lactate compared to the one-plasmid strain *A. woodii* P_*bgaL*__LDHD. Comparing the D-lactate production rates *A. woodii* P_*bgaL*__LDHD_P_*ackA-*theo__PFL showed lower production rates than *A. woodii* P_*bgaL*__LDHD.

All strains showed growth and acetate formation. *A. woodii* P_*bgaL*__LDHD and *A. woodii* P_*bgaL*__LDHD_P_*ackA-*theo__PFL additionally showed lactate formation. The highest maximum lactate production of 16.58 ± 1.32 mM was reached by *A. woodii* P_*bgaL*__LDHD (lactose-induced) after 72 h of cultivation, followed by *A. woodii* P_*bgaL*__LDHD_P_*ackA-*theo__PFL (lactose- and theophylline-induced) with a maximum D-lactate concentration of 11.58 ± 6.32 mM after 56 h. The control experiments using non-induced *A. woodii* P_*bgaL*__LDHD_P_*ackA-*theo__PFL and *A. woodii* WT cells yielded with 3.6 ± 1.2 mM comparable low or no D-lactate concentrations, respectively (Fig. 5b). Surprisingly, after 60 h, the D-lactate concentration of *A. woodii* P_*bgaL*__LDHD_P_*ackA-*theo__PFL decreased. The maximum D-lactate titers of the respective strains were normalized to OD_600_ for a biomass-based comparison. Thus, after induction, *A. woodii* P_*bgaL*__LDHD (lactose-induced) and *A. woodii* P_*bgaL*__LDHD_P_*ackA-*theo__PFL (lactose- and theophylline-induced) reached a D-lactate concentration of 21.63 ± 2.87 mM OD_600_^−1^ and 18.26 ± 10.09 mM OD_600_^−1^, respectively (Table 3). Complementary, a molar D-lactate:acetate ratio of 0.10 ± 0.01 was achieved with the lactose-induced *A. woodii* P_*bgaL*__LDHD cells, whereas the lactose- and theophylline-induced *A. woodii* P_*bgaL*__LDHD_P_*ackA-*theo__PFL cells showed a molar D-lactate:acetate ratio of 0.08 ± 0.01 (Table 3).

**Table 3:** Maximum lactate concentration, lactate:acetate ratio, and lactate production rates after induction of *A. woodii* P_*bgaL*__LDHD (lactose-induced) and *A. woodii* P_*bgaL*__LDHD_P_*ackA*-theo__PFL (lactose- and theophylline-induced) achieved in autotrophic growth experiments in serum bottles, the related *p*-values with − marking non-significant differences and + marking significant differences

	***A. woodii* P**_***bgaL***_**_LDHD**	***A. woodii* P**_***bgaL***_**_LDHD_P**_***ackA-*theo**_**_PFL**	***p-value***
Maximum lactate concentration post-induction [mM]	16.58 ± 1.32	11.58 ± 6.32	0.09 (−)
Maximum lactate/acetate post-induction [−]	0.10 ± 0.01	0.08 ± 0.01	0.01 (+)
Maximum lactate/formate post-induction [−]	4.33 ± 0.79	1.16 ± 0.29	0.00 (+)
Maximum produced lactate concentration post-induction normalized to OD_600_ 1 [mM · OD_600_^−1^]	21.6 ± 2.9	18.3 ± 10.1	0.29 (−)
Maximum lactate production rate post-induction [mM · h^−1^]	0.85 ± 0.24	1.43 ± 0.77	0.11 (−)

As already described by Mook et al. (2022), formate production was also detected in this study with *A. woodii* P_*bgaL*__LDHD with a maximum of 8.49 ± 1.45 mM after 72 h. Lactose- and theophylline-induced *A. woodii* P_*bgaL*__LDHD_P_*ackA-*theo__PFL cells showed a maximum formate concentration of 21.40 ± 5.26 mM (Fig. 5e). The maximum formate concentration after induction produced by *A. woodii* P_*bgaL*__LDHD (lactose-induced) and *A. woodii* P_*bgaL*__LDHD_P_*ackA-*theo__PFL (lactose- and theophylline-induced) amounted to 7.95 ± 1.58 mM and 8.16 ± 7.37 mM, respectively.

*A. woodii* WT and the control strain *A. woodii* P_*bgaL*__LDHD_P_*ackA-*theo__PFL (not induced) reached maximum OD_600_ values of 1.33 ± 0.16 and 0.98 ± 0.22, respectively. In contrast, the maximum OD_600_ was lower than with the induced *A. woodii* P_*bgaL*__LDHD and *A. woodii* P_*bgaL*__LDHD_P_*ackA-*theo__PFL strains (0.88 ± 0.04 and 0.81 ± 0.02, respectively). After induction, the growth of both strains stopped (Fig. 5a).

During cultivation in serum bottles, a reduction in pH was evident for all strains, whereas *A. woodii* WT reached the strongest decrease to pH 5.34 ± 0.06. In contrast, a decrease to pH 6.01–6.35 was detected with the recombinant *A. woodii* strains (Fig. 5f). The highest total gas consumption was recorded with *A. woodii* WT with 1,967 ± 104 hPa. The recombinant *A. woodii* strains showed similar gas uptakes in a range of 1100–1433 hPa (Fig. 5d).

The main product of all *A. woodii* strains was acetate. *A. woodii* WT displayed its highest acetate concentration of 230 ± 24.7 mM after 72 h. *A. woodii* P_*bgaL*__LDHD (induced) displayed its maximum acetate concentration (161 ± 18.8 mM) after 72 h, while *A. woodii* P_*bgaL*__LDHD_P_*ackA-*theo__PFL (induced and not induced) reached their acetate maximum after 56 h or 72 h with 150 ± 66.2 mM and 173 ± 56.2 mM, respectively (Fig. 5c).

*A. woodii* P_*bgaL*__LDHD showed a significantly higher maximum molar D-lactate:formate ratio (*p* = 0.01) as well as a significantly higher D-lactate:acetate ratio (*p *= 0.01, Table 3).

### Amended fermentation profile caused by promoter exchange

In the following autotrophic growth experiments in serum bottles, all engineered strains expressing the *ldhD* gene under control of the P_*lctA*_ promoter grew and produced acetate as well as D-lactate. These strains showed an increase in D-lactate production compared to the respective strains carrying the lactose-inducible P_*bgaL*_ promoter system. In combination with P_*lctA*_, regulating the *ldhD* expression, higher D-lactate titers were achieved by additionally expressing *pflA* and *pflB* recombinantly.

**Fig. 6:**
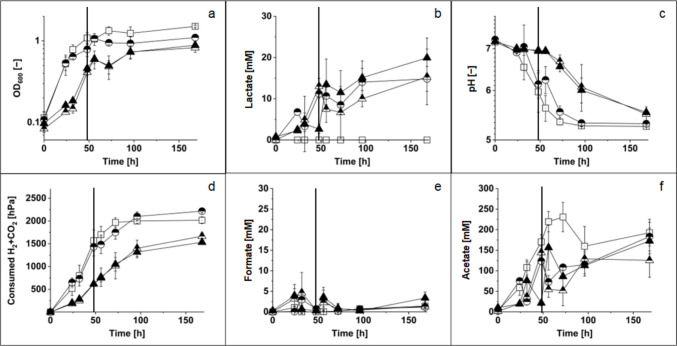
Autotrophic growth experiments in serum bottles with the strains *A. woodii* WT (white squares), *A. woodii* P_*lctA*__LDHD lactate-induced (half-filled circles), *A. woodii* P_*lctA*__LDHD_P_*ackA-*theo__PFL lactate-induced (half-filled triangles), and *A. woodii* P_*lctA*__LDHD_P_*ackA-*theo__PFL lactate- and theophylline-induced (black triangles). OD_600_ (**a**), D-lactate concentration (**b**), pH (**c**), consumption of H_2_ and CO_2_ (**d**), formate concentration (**e**), and acetate concentration (**f**) were monitored. The full line shows the induction of the *A. woodii* P_*lctA*__LDHD_P_*ackA-*theo__PFL cells with 1 mM theophylline. *n* = 3. Error bars indicate standard deviations

No external lactate was used to induce the *ldhD* expression due to leaky expression of the P_*lctA*_ promoter system. *A. woodii* P_*lctA*__LDHD produced a maximum D-lactate concentration of 16.49 ± 1.69 mM after 168 h (Fig. 6b). Normalized to an OD_600_ of 1, this corresponds to a maximum D-lactate concentration of 18.01 ± 5.12 mM OD_600_^−1^ (Table 4). The *A. woodii* P_*lctA*__LDHD_P_*ackA-*theo__PFL cells (not additionally induced with theophylline) produced a similar maximum concentration of 16.99 ± 5.05 mM D-lactate. *A. woodii* P_*lctA*__LDHD_P_*ackA-*theo__PFL (additionally theophylline-induced) produced a maximum of 21.00 ± 2.8 mM D-lactate after 168 h. Normalized to an OD_600_ of 1, this D-lactate concentration is equivalent to 22.43 ± 3.76 mM OD_600_^−1^ (Table 4). The maximum molar D-lactate:acetate ratio was 0.11 ± 0.02 and 0.13 ± 0.01 with *A. woodii* P_*lctA*__LDHD (lactate-induced) and *A. woodii* P_*lctA*__LDHD_P_*ackA-*theo__PFL (lactate- and theophylline-induced), respectively.

**Table 4:** Maximum D-lactate concentration, molar D-lactate:acetate ratio, specific D-lactate concentration, and D-lactate production rate of *A. woodii* P_*lctA*__LDHD (D-lactate induced) and *A. woodii* P_*lctA*__LDHD_P_*ackA-*theo__PFL (D-lactate- and theophylline-induced) achieved in autotrophic growth experiments in serum bottles, the related *p*-values with − marking non-significant differences and + marking significant differences; n.d. = (not determinable, because no formate was detected or below quantification limit, in at least one triplicate)

	***A. woodii* P**_***lctA***_**_LDHD**	***A. woodii* P**_***lctA***_**_LDHD_**P_***ackA-*theo**__**PFL**	***p-value***
Maximum produced D-lactate [mM]	16.49 ± 1.69	21.00 ± 2.41	0.01 (+)
Maximum molar D-lactate/acetate [−]	0.11 ± 0.02	0.13 ± 0.01	0.06 (−)
Maximum molar D-lactate/formate post-induction [−]	n.d	n.d	n.d
Maximum produced D-lactate normalized to OD_600_ [mM · OD_600_^−1^]	18.01 ± 5.12	22.43 ± 3.76	0.11 (−)
Maximum D-lactate production rate [mM · h^−1^]	0.49 ± 0.12	1.48 ± 0.68	0.01 (+)

During growth, *A. woodii* P_*lctA*__LDHD reached a maximum OD_600_ of 1.10 ± 0.09 (Fig. 6a). The theophylline-induced *A. woodii* P_*lctA*__LDHD_P_*ackA-*theo__PFL cells and the theophylline-induced *A. woodii* P_*lctA*__LDHD_P_*pta-ack*__PFL cells reached a peak OD_600_ of 0.88 ± 0.03 and 0.82 ± 0.10, respectively (Fig. 6a), which hardly differs from the OD_600_ reached by *A. woodii* P_*bgaL*__LDHD_P_*ackA-*theo__PFL (lactose- and theophylline-induced and non-induced) in the respective batch experiment. In contrast, *A. woodii* WT reached a maximum OD_600_ of 1.50 ± 0.05.

Acetate was still the main metabolic product produced by all strains. *A. woodii* WT produced its maximum acetate concentration after 72 h (230.7 ± 35.8 mM). The *A. woodii* P_*lctA*__LDHD produced 20% less acetate, with a peak acetate concentration of 183.2 ± 34.7 mM at the end of the cultivation, followed by *A. woodii* P_*lctA*__LDHD_P_*ackA-*theo__PFL (additionally theophylline-induced) with a maximum acetate concentration of 172.6 ± 31.0 mM. The lowest acetate concentration was produced by *A. woodii* P_*lctA*__LDHD_P_*ackA-*theo__PFL (not theophylline-induced) with 144.3 ± 16.0 mM (Fig. 6c).

With the P_*lctA*_ promoter, formate was produced in lower concentrations. *A. woodii* P_*lctA*__LDHD displayed a formate concentration of only 34% of the concentration reached with *A. woodii* P_*bgaL*__LDHD. *A. woodii* P_*lctA*__LDHD_P_*ackA-*theo__PFL displayed only 22% of the formate produced with *A. woodii* P_*bgaL*__LDHD_P_*ackA-*theo__PFL (Fig. 6e). *A. woodii* WT produced 1.4 ± 0.3 mM formate at the end of this experiment (Fig. 6e). *A. woodii* P_*lctA*__LDHD produced its maximum formate concentration after 32 h (3.0 ± 1.5 mM). The formate concentration produced by the lactate-induced *A. woodii* P_*lctA*__LDHD_P_*ackA-*theo__PFL cells fluctuated during the growth experiment and reached its maximum concentration after 32 h (4.7 ± 4.9 mM). *A. woodii* P_*lctA*__LDHD_P_*ackA-*theo__PFL (lactate- and theophylline-induced), as well as *A. woodii* P_*lctA*__LDHD_P_*ackA-*theo__PFL (lactate-induced), showed a fluctuating formate concentration during cultivation with a maximum of 4.0 ± 2.7 mM after 24 h (Fig. 6e).

With the lactate-induced *A. woodii* P_*lctA*__LDHD and *A. woodii* WT, the pH dropped until around 100 h and stayed stable during further cultivation (Fig. 6f). The pH during cultivation of *A. woodii* P_*lctA*__LDHD_P_*ackA-*theo__PFL (theophylline-induced and not theophylline- induced) dropped to pH 5.5 after around 150 h and remained stable during further batch cultivation in serum bottles.

Regarding the consumed H_2_ and CO_2_, *A. woodii* WT showed an overall pressure loss of 2017 ± 76.4 hPa in this batch experiment (Fig. 6d). The total pressure loss of *A. woodii* P_*lctA*__LDHD (lactate-induced) amounted to 2217 ± 28.9 hPa (Fig. 6d). *A. woodii* P_*lctA*__LDHD_P_*ackA-*theo__PFL (lactate-induced) showed a pressure loss of 1667 ± 76.4 hPa H_2_ and CO_2_, while *A. woodii* P_*lctA*__LDHD_P_*ackA-*theo__PFL (lactate- and theophylline-induced) resulted in a total pressure loss of 1533 ± 28.9 hPa (Fig. 6d).

*A. woodii* P_*lctA*__LDHD_P_*ackA-*theo__PFL significantly produced the highest maximum absolute amount of D-lactate (*p* = 0.01) and displayed also a significantly higher maximum D-lactate production rate (*p* = 0.01) compared to *A. woodii* P_*lctA*__LDHD (Table 4).

Since these bottle-scale results were promising, the setup was scaled up to a stirred tank reactor (STR) with continuous gassing.

### Batch cultivation of the P_*lctA*_ promoter *A. woodii* strains in a stirred tank bioreactor with continuous gassing

Autotrophic batch cultivations were performed with the strains *A. woodii* P_*lctA*__LDHD and *A. woodii* P_*lctA*__LDHD_P_*ackA-*theo__PFL in a controlled STR with continuous gassing. *A. woodii* P_*lctA*__LDHD_P_*ackA-*theo__PFL produced 34.9 mM D-lactate in the STR.

**Fig. 7:**
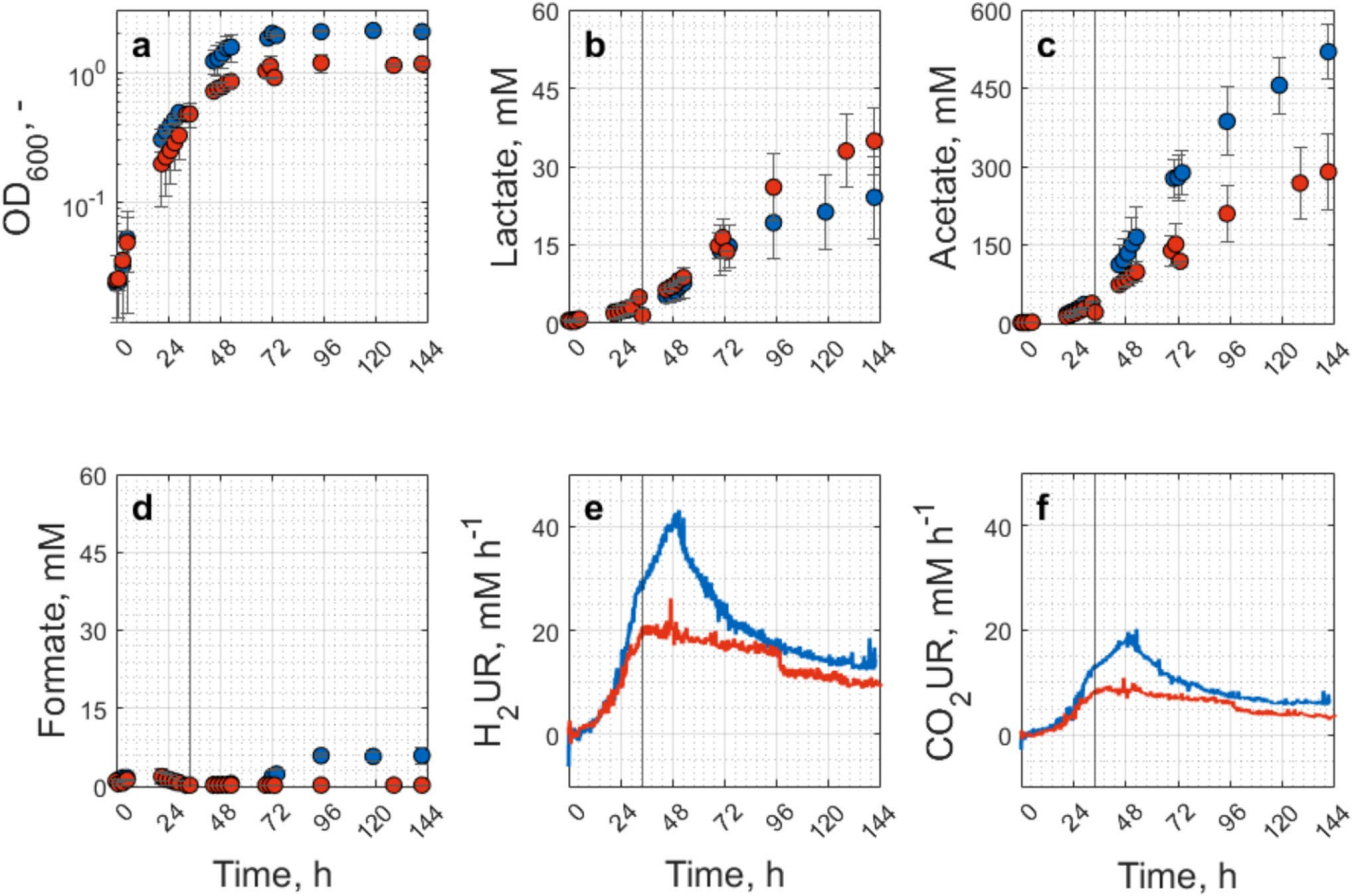
Autotrophic batch cultivations of *A. woodii* P_*lctA*__LDHD D-lactate-induced (blue circles) and *A. woodii* P_*lctA*__LDHD_P_*ackA-*theo__PFL D-lactate- and theophylline-induced (red circles) in a STR with continuous gassing. The black line marks the induction time point of the PFL genes with 1 mM theophylline. OD_600_ (**a**), D-lactate concentration (**b**), acetate concentration (**c**), formate concentration (**d**), H_2_ uptake rate (**e**), and CO_2_ uptake rate (**f**) were monitored gas (*T* = 30 °C; pH = 7.0; *P V*^−1^ = 6.06 W L^−1^, *F*_gas_ = 0.08 vvm, *n* = 2). Error bars indicate minimum and maximum values measured. For gas uptake rates, one representative run is displayed for each strain

The P_*lctA*_ promoter was not externally induced. *A. woodii* P_*lctA*__LDHD_P_*ackA-*theo__PFL was induced with 1 mM theophylline at an OD_600_ of 0.6 (Fig. 7).

Growth of *A. woodii* P_*lctA*__LDHD_P_*ackA-*theo__PFL was continuously reduced compared to *A. woodii* P_*lctA*__LDHD already after process start (Fig. 7a). The maximum OD_600_ measured were 2.10 after 119 h with *A. woodii* P_*lctA*__LDHD and 1.19 after 95 h with *A. woodii* P_*lctA*__LDHD_P_*ackA-*theo__PFL (Table 5). The latter represents a decrease of 46% compared to *A. woodii* P_*lctA*__LDHD. After and prior to reaching the maximum, OD_600_ remained more or less stable after the exponential growth phases with both strains.

**Table 5:** Maximum D-lactate concentration, molar D-lactate:acetate ratio, specific D-lactate concentration, and D-lactate production rate during autotrophic batch cultivation of *A. woodii* P_*lctA*__LDHD (lactate-induced) and *A. woodii* P_*lctA*__LDHD_P_*ackA-*theo__PFL (lactate- and theophylline-induced) in a STR with continuous gassing; maximum ratios were determined after the lag-phase; n.d. = not determinable, because no formate was detected or below quantification limit, in at least one duplicate; percentages given show the deviation from the respective serum bottle cultivation; *n* = 2

	***A. woodii* P**_***lctA***_**_LDHD**		***A. woodii* P**_***lctA***_**_LDHD_P**_***ackA-*theo**_**_PFL**	
Maximum produced D-lactate [mM]	24.07	+ 46%	34.92	+ 66%
Maximum molar D-lactate/acetate ratio [−]	0.12	+ 7%	0.13	+ 1%
Maximum molar D-lactate/formate ratio post-induction [−]	22.96	n.d	n.d	n.d
Maximum produced D-lactate normalized to OD_600_ [mM · OD_600_^−1^]	11.66	− 35%	29.87	+ 33%
Maximum produced biomass-specific D-lactate [mmol g^−1^]	23.80	− 35%	60.96	+ 33%
Maximum D-lactate production rate [mM h^−1^]	0.42	− 13%	0.80	− 46%

Both *A. woodii* strains produced D-lactate. After 141 h, *A. woodii* P_*lctA*__LDHD reached a maximum D-lactate concentration of 24.07 mM. *A. woodii* P_*lctA*__LDHD_P_*ackA-*theo__PFL produced 34.92 mM D-lactate (Fig. 7b). This corresponds to 11.66 mM OD_600_^−1^ (23.80 mmol g_CDW_^−1^) and 29.88 mM OD_600_^−1^ (60.96 mmol g_CDW_^−1^), respectively. Regarding the absolute concentrations, *A. woodii* P_*lctA*__LDHD_P_*ackA-*theo__PFL produced 45% more D-lactate compared to *A. woodii* P_*lctA*__LDHD. The maximum molar D-lactate/acetate ratio was 0.12 with *A. woodii* P_*lctA*__LDHD and 0.13 with *A. woodii* P_*lctA*__LDHD_P_*ackA-*theo__PFL. The maximum D-lactate production rates were 0.42 mM h^−1^ and 0.80 mM h^−1^, respectively. Notably, during cultivation of *A. woodii* P_*lctA*__LDHD_P_*ackA-*theo__PFL, this rate was reached after induction of the PFL plasmid within 71 h, while the single plasmid strain reached the maximum rate after 51 h. Compared to the serum bottle cultivations, the maximum absolute D-lactate concentrations were increased by 46% with *A. woodii* P_*lctA*__LDHD and 66% with *A. woodii* P_*lctA*__LDHD_P_*ackA-*theo__PFL, respectively.

Also, during controlled STR cultivations with continuous gassing, acetate remained the main product. Maximum acetate concentrations were reached at the end of the batch cultivations and amounted to 519.69 mM with *A. woodii* P_*lctA*__LDHD and 290.02 mM with *A. woodii* P_*lctA*__LDHD_P_*ackA-*theo__PFL, respectively (Fig. 7c). The maximum OD_600_ specific acetate concentration is nearly similar in both strains: *A. woodii* P_*lctA*__LDHD reached 251.71 mM OD_600_^−1^ (513.76 mmol g_CDW_^−1^), and *A. woodii* P_*lctA*__LDHD_P_*ackA-*theo__PFL reached 248.10 mM OD_600_^−1^ (506.32 mmol g_CDW_^−1^).

Both strains showed an initial increase and subsequent decrease in formate concentrations in the batch processes (Fig. 7d). Initial peaks were obtained at 1.82 mM with *A. woodii* P_*lctA*__LDHD and 1.88 mM after 21 h with *A. woodii* P_*lctA*__LDHD_P_*ackA-*theo__PFL, respectively. Subsequently, accumulated formate was nearly reconsumed by both strains. During cultivation of *A. woodii* P_*lctA*__LDHD, formate concentrations started to increase again after 71 h of cultivation time, reaching a plateau after 95 h at ~5.90 mM. Formate concentrations did not increase again with *A. woodii* P_*lctA*__LDHD_P_*ackA-*theo__PFL.

Matching biomass data, the maximum gas uptake rates during cultivation of *A. woodii* P_*lctA*__LDHD were strongly increased compared to *A. woodii* P_*lctA*__LDHD_P_*ackA-*theo__PFL. Maximum H_2_ uptake rates were 43.12 mM h^−1^ (after 51 h) with *A. woodii* P_*lctA*__LDHD, but only 26.15 mM h^−1^ (after 47 h) with *A. woodii* P_*lctA*__LDHD_P_*ackA-*theo__PFL (Fig. 7e). At the H_2_ peak, *A. woodii* P_*lctA*__LDHD_P_*ackA-*theo__PFL consumed only 60% of the H_2_ taken up by *A. woodii* P_*lctA*__LDHD. Maximum CO_2_ uptake rates were 20.26 mM h^−1^ (after 53 h) with *A. woodii* P_*lctA*__LDHD and 10.90 mM h^−1^ (after 47 h) with *A. woodii* P_*lctA*__LDHD_P_*ackA-*theo__PFL, respectively (Fig. 7f). At the time point of the maximum H_2_ uptake rate, the measured molar H_2_:CO_2_ consumption ratios were 2.65 (*A. woodii* P_*lctA*__LDHD) and 2.40 (*A. woodii* P_*lctA*__LDHD_P_*ackA-*theo__PFL).

**Fig. 8:**
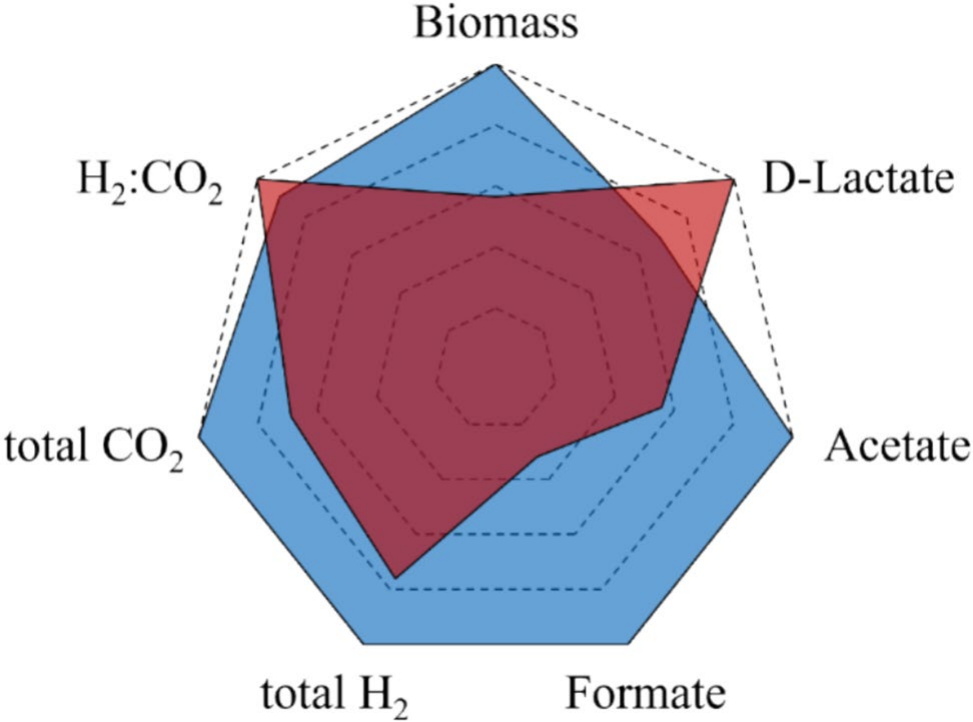
Comparison of maximum biomass, D-lactate, acetate, and formate concentrations, as well as relative total CO_2_ and H_2_ uptake, and total H_2_:CO_2_ uptake ratios during autotrophic batch cultivations of *A. woodii* P_*lctA*__LDHD (blue) and *A. woodii* P_*lctA*__LDHD_P_*ackA-*theo__PFL (red) in the STR with continuous gassing

Selected process performance indicators are summarized in Fig. 8, comparing both strains. STR batch processes with *A. woodii* P_*lctA*__LDHD_P_*ackA-*theo__PFL with continuous gassing yielded the highest D-lactate concentration (Fig. 8). *A. woodii* P_*lctA*__LDHD displayed higher biomass, acetate, and formate concentrations in combination with higher total CO_2_ and H_2_ consumption. However, the *A. woodii* strain with improved D-lactate production consumed a little bit more H_2_ compared to CO_2_. Carbon balances were closed within the estimation error to 98% (*A. woodii* P_*lctA*__LDHD) and 104% (*A. woodii* P_*lctA*__LDHD_P_*ackA-*theo__PFL), respectively. Electron balances showed a slightly reduced recovery between 93 and 94%.

## Discussion

The global demand for lactate continues to grow steadily. In 2024, the lactate market was valued at USD 3454.6 million. By 2033, it is projected to reach USD 6653.7 million, with a compound annual growth rate (CAGR) of 7.7% from 2025 to 2033 (Grand View Research 2025). Lactic acid is classified as GRAS (generally regarded as safe) and can therefore be used in food and cosmetic manufacturing (Abedi and Hashemi 2020). It is primarily produced through fermentation from plant-based sugars or starch-containing substrates (Abedi and Hashemi 2020; Ghaffar et al. 2014). The use of plants as a feedstock for lactate production leads to a conflict in land use. Lactate produced from gas fermentation or from liquid C1 compounds offers a more sustainable and conflict-free option to serve the growing market.

In this study, we demonstrated improved D-lactate production with recombinant *A. woodii* strains by exchanging the promoter system, introducing a two-plasmid architecture, and expressing PFL.

The lactose-inducible P_*bgaL*_ promoter is a strong promoter system in *A. woodii* (Beck 2020). In this study, it was observed that all strains carrying P_*bgaL*_ to regulate *ldhD* gene expression and cultivated in serum bottles, stopped growing immediately after the induction of the cells with lactose, corresponding with a sudden and rapid start of D-lactate production (Fig. 5).

In contrast, the lactate-inducible P_*lctA*_ promoter originates from *A. woodii* itself (Schoelmerich et al. 2018). All strains carrying P_*lctA*_ to control LDHD expression exhibited slower and continuous D-lactate production (Fig. 6), while still growing and building up biomass. The slow and continuous D-lactate production indicates a slowly increasing LDHD availability, which can be ascribed to the leaky expression of the *ldhD* gene due to the lactate-inducible promoter system P_*lctA*_. The continuous growth of the *A. woodii* strains shows that the cells are still able to produce D-lactate during anabolism. Since these cells were never externally induced with lactate, a self-induction due to the leakiness of this promoter system happened. P_*lctA*_ likely provides graded induction, preventing a metabolic shock and enabling the cells to maintain anabolic activity while producing D-lactate. In *E. coli*, Bienick et al. (2014) observed a linear correlation between higher promoter activity and increased protein expression, as well as a linear correlation between decreasing microbial growth rates and increased heterologous protein expression. They concluded for *E. coli*, the gene expression cost can restrict the pathway flux in metabolic engineering. Additionally, carrying a plasmid and producing recombinant proteins are burdens to cells (Borkowski et al. 2016). In *E. coli* RR1, it was observed that an increase in plasmid copy number, as well as the expression of a heterologous protein, was accompanied by a decrease in the growth rate of the cells (Bentley et al. 1990).

In this study, the cellular burden imposed by P_*bgaL*_ becomes particularly evident when comparing plasmid-based expression with genome integration. Genome integration of the *ldhD* gene regulated by P_*bgaL*_ resulted in decreased D-lactate production compared to the reference strain *A. woodii* P_*bgaL*__LDHD (Figures S2, S3, S4). This suggests that the lower gene copy number leads to less LDHD availability. These findings demonstrate that high promoter strength combined with high copy numbers (plasmid) pushes the cells into catabolism, whereas a low copy number (genome integration) provides insufficient LDHD levels for high D-lactate production.

A further enhancement was achieved with the establishment of a two-plasmid system in *A. woodii.* One plasmid carrying the *ldhD* gene and its respective promoter system, and a second plasmid carrying the *pflB* gene originated from *C. pasteurianum*, regulated by the theophylline-inducible promoter system P_*ackA*-theo_ (Beck 2020) and the associated *pflA* gene controlled by P_*pta-ack*_ originated from *C. ljungdahlii*. Our results demonstrated a benefit in D-lactate production due to the additional expression of the PFL in *A. woodii* in serum bottles as well as under continuously gassed conditions in a stirred tank bioreactor (Figs. 5 and 6, Tables 4 and 5). The PFL is a homo-dimer and belongs to the glycyl radical enzymes (Becker et al. 1999; Crain and Broderick 2013). Especially during anabolism, bacterial cells have a higher need for amino acids and polysaccharides. Pyruvate is a central metabolite for the production of these components. Natively, the enzyme PFOR performs pyruvate synthesis from acetyl-CoA and CO_2_, while oxidizing Fd^2−^ (Blamey and Adams 1993). In the recombinant *A. woodii* P_*lctA*__LDHD_P_*ackA-*theo__PFL cells, it is likely assumed that pyruvate is also produced by the recombinantly expressed PFL, albeit direct proof is pending. Since the Gibbs energy of the PFOR reaction (Δ_r_*G*′^m^ = 18.0 ± 13.1 kJ mol⁻^1^) and the PFL reactions (Δ_r_*G*′^m^ = 19.8 ± 2.9 kJ mol⁻^1^) towards pyruvate are on a similar level, and the PFOR requires a Fd^2−^ per pyruvate molecule and the PFL does not, the PFL reaction would be an advantage during anabolism, saving reduction equivalents. The saved Fd^2−^ molecule could be channeled to the Rnf complex, which reduces one NAD^+^ to NADH (Westphal et al. 2018). The benefit in D-lactate production with the PFL activity during anabolism makes this NADH available for the conversion of pyruvate to D-lactate via LDHD (Li et al. 2012). Since PFOR and PFL show similar Gibbs free energies for the reaction towards pyruvate, it is assumed that both enzymes work in parallel, and the PFL reaction does not replace the PFOR reaction. However, this could not be proven in this study.

*A. woodii* also has a native PFL and activating enzyme. These enzymes were recently recombinantly expressed in *C. ljungdahlii* and led to comparable results as observed in this study, when establishing *C. pasteurianum*’s PFL in *A. woodii*. Im et al. (2025) observed an increase in growth and acetate production if a formate excess was supplemented to the substrates H_2_ and CO_2_ (Im et al. 2025). The achieved growth and production enhancement were explained by the higher pyruvate availability due to the PFL activity in the presence of sufficient amounts of formate.

The induction of LDHD caused by the strong promoter system P_*bgaL*_ resulted in a metabolic switch from anabolism to catabolism in *A. woodii* P_*bgaL*__LDHD_P_*ackA-*theo__PFL cells, and the recombinantly expressed PFL showed no benefit in D-lactate production but led to a further increase in formate accumulation (Fig. 3, Table 3). This suggests that the PFL catalyzes in the *A. woodii* P_*bgaL*__LDHD_P_*ackA-*theo__PFL cells the production of formate and acetyl-CoA from pyruvate, which is the enzyme’s thermodynamically preferred reaction. Especially, during catabolism, most of the acetyl-CoA was converted to acetate via acetyl-phosphate by the enzymes phosphotransacetylase and acetate kinase. The acetate kinase generates one ATP per acetate during the conversion from acetyl-phosphate to acetate. Formate is continuously produced via HDCR. If most of the acetyl-CoA is used for ATP generation via acetate production, not enough ATP was available to convert all formate to formyl-THF and subsequently formate accumulated. When acetyl-CoA is mainly turned into acetate, the threshold of acetyl-CoA (*K*_m_ = 0.051 mM, Knappe et al. 1974) for converting formate and acetyl-CoA to pyruvate and CoA may no longer be achievable. This leads to a formate accumulation due to production by HDCR and by PFL.

In general, D-lactate could be successfully produced with *A. woodii* P_*lctA*__LDHD and *A. woodii* P_*lctA*__LDHD_P_*ackA-*theo__PFL in the STR with continuous gassing. In line with results from cultivations in serum bottles, the batch process with *A. woodii* P_*lctA*__LDHD_P_*ackA-*theo__PFL in the STR resulted in reduced biomass concentrations in combination with increased D-lactate concentrations compared to the batch process with *A. woodii* P_*lctA*__LDHD. In general, both strains, *A. woodii* P_*lctA*__LDHD and *A. woodii* P_*lctA*__LDHD_P_*ackA-*theo__PFL, reached higher D-lactate concentrations in the STR compared to serum bottles. However, acetate formation was increased, and formate accumulation was reduced in the batch process with *A. woodii* P_*lctA*__LDHD_P_*ackA-*theo__PFL in the STR compared to serum bottles. The reason for this may lie in the improved availability of H_2_ due to increased power inputs and continuous gassing in the STR. Increased dissolved H_2_ potentially enabled a higher supply of reducing equivalents as more H_2_ was available for hydrogenases to convert to NADH and Fd_red_. However, dissolved H_2_ concentrations and intracellular redox equivalents were not directly measured in this study. The reduced formate accumulation in *A. woodii* P_*lctA*__LDHD_P_*ackA-*theo__PFL indicates that the PFL operated in the pyruvate-forming direction under these conditions. The single-plasmid strain, however, accumulated formate in the STR, suggesting that formate could not be further metabolized under gas-saturated and likely more catabolic conditions without the additional pyruvate supply provided by the PFL. Interestingly, although the final acetate concentration was higher in the batch process with *A. woodii* P_*lctA*__LDHD, biomass-specific acetate concentrations were similar between the two strains. Possibly, under gas-saturated conditions, acetate formation in *A. woodii* is mainly governed by the WLP-derived acetyl-CoA availability and the ATP requirements for growth. Therefore, it would become largely independent of the intracellular pyruvate pool. Under these conditions, the WLP and energy conservation system activity are high, potentially leading to increased acetyl-CoA formation rates and therefore increased biomass formation compared to serum bottle experiments. This could explain why the biomass-specific D-lactate concentrations of the single-plasmid strain were lower compared to serum bottle cultivations. In contrast, in the two-plasmid system, accumulating acetyl-CoA may be partially converted by the PFL into pyruvate and subsequently D-lactate, which could account for the increased overall and biomass-specific D-lactate concentrations with *A. woodii* P_*lctA*__LDHD_P_*ackA-*theo__PFL, despite similar biomass-specific acetate levels. Carbon and electron balances were closed within the estimation error in the STR cultivations, indicating reliable process results. *A. woodii* P_*lctA*__LDHD_P_*ackA-*theo__PFL directs a larger share of fixed carbon and electrons into lactate (with reduced acetate formation), compared to *A. woodii* P_*lctA*__LDHD.

Also, other studies have reached out to produce lactate using *A. woodii* as well. A non-plasmid-based approach to produce lactate was shown by Moon et al. (2023). Moon et al. (2023) produced 20.4 ± 0.5 mM lactate with resting *A. woodii ∆hydBA/hdcr* double mutant cells. The genes encoding *A. woodii*’s hydrogenases were deleted to redirect the electron flux. Additionally, this reversed the reaction of *A. woodii*’s native LctBCD complex, producing lactate. The *A. woodii ΔhydBA/hdcr* mutant was no longer able to grow with H_2_ and CO_2_ but produced D-lactate and acetate from glycine betaine and CO (Moon et al. 2023). Herzog et al. (2023) used a plasmid-based approach with the plasmid pMTL83251_P_*bgaL*__NFP (Mook et al. 2022) to produce lactate with *A. woodii*. They were able to produce appr. 90 mM D-lactate using a STR, but with continuous H₂ + CO₂ gassing and 6 g L⁻^1^ yeast extract. Stock et al. (2026) demonstrated successfully that changes in the process variables also increase the D-lactate production with *A. woodii*. The approach was to supplement small amounts of CO to the gas flow during continuous H_2_ + CO_2_ gassing of *A. woodii* P_*lctA*__LDHD to increase the availability of reduction equivalents in *A. woodii* and thereby increase biomass formation and D-lactate production. This idea resulted in an 189% increased lactate production (Stock et al. 2026).

In this study, an *A. woodii* strain, *A. woodii* P_*lctA*__LDHD_P_*ackA-*theo__PFL, was demonstrated, which was capable of producing up to 34.9 mM D-lactate besides its main product, acetate, only using H_2_ + CO_2_. A possible usage of this strain is cultivating together with a chain-elongating bacterium in a synthetic lactate-mediated co-culture, producing mid-chain fatty acids. Since the strain performed that well in a 2 L STR, it shows potential for further upscaling in larger bioreactors.

## Conclusion

The D-lactate production using a two-plasmid system in *A. woodii* P_*lctA*__LDHD_P_*ackA-*theo__PFL, shown in this study, turned out to be a well-performing strain for producing lactate in batch cultivations from H_2_ and CO_2_. The establishment of PFL and PFL-AE, as a second pyruvate-gaining enzyme in addition to the PFOR in *A. woodii* P_*lctA*__LDHD, resulted in a clear increase in D-lactate titers. This new way of D-lactate production offers an opportunity to recycle industrial waste gas and produce higher-value products, such as D-lactate from it. Using a strong promoter system, such as the lactose-inducible P_*bgaL*_ promoter, led to a stop in cell growth after induction of cells. Once this was avoided by changing the promoter system to the leaky, lactate-inducible P_*lctA*_ promoter, *A. woodii* still grew after induction. D-Lactate was produced more slowly but continuously during the growth, and higher D-lactate concentrations were reached. A genome integration of the *ldhD* gene achieved no benefit in D-lactate production with *A. woodii*.

## Supplementary Information

Below is the link to the electronic supplementary material.

ESM 1 (DOCX 936 KB)

## Data Availability

The datasets generated during and/or analysed during the current study are available from the corresponding author on reasonable request.
