# Supplementary material for: Improving autotrophic D-lactate production by heterologous expression of a pyruvate formate lyase in an LDHD-expressing Acetobacterium woodii strain
Source: Appl Microbiol Biotechnol. 2026 Jul 10;110(1):215. doi: 10.1007/s00253-026-13952-5 (PMC13372834; doi:10.1007/s00253-026-13952-5)
Supplement: Supplementary file 1 — (DOCX 936 KB) [file 253_2026_13952_MOESM1_ESM.docx]

**Supplementary**

**Table S1.** Plasmids and primers used in this work.

| Plasmid | Purpose and description | Construction and verification primer | Source |
| --- | --- | --- | --- |
| pMTL-MPD21 | -Deletion of the *pheA* gene  -Plasmid contains CRISPR array, homology arms displaying the pheA deletion, and a chloramphenicol/ thiamphenicol resistance gene *catP* | FW_pheAHA1.5_out: CCGGAAA GGGATCAAATTTG  RV_pheAHA1.5_out: TGGTTTC GTCTTTTTTAGGTAG  ColE1+tra-F2: CCATCAAGA AGAGCGAC  pCD6-R1: GACTTTAAG CCTACGAATACC | [Poulalier-Delavelle](https://www.researchgate.net/scientific-contributions/Margaux-Poulalier-Delavelle-2254576711?_tp=eyJjb250ZXh0Ijp7ImZpcnN0UGFnZSI6Il9kaXJlY3QiLCJwYWdlIjoicHVibGljYXRpb24iLCJwcmV2aW91c1BhZ2UiOiJfZGlyZWN0In19) et al., 2023 |
| pMTL-MPD23_P*_bgaL_*_ldhD | -Reconstruction of the *pheA* gene and genome integration of the *ldhD* gene regulated by the P*_bgaL_* promoter system  -Plasmid contains: CRISPR array, homology arms displaying the pheA gene and its genomic environment, flanking the P*_bgaL_* promoter and the *ldhD* gene, and a chloramphenicol/ thiamphenicol resistance gene *catP* | P*_bgaL_*_fwd: TAACCCCGGATCCGAGTCGCTCTAGATAATTTAGATATTAATTCTAAATTAAGTGAAATTAATATAG  P*_bgaL_*_rev: AAATCTTCATACCCTCCCAATACATTTAAAATAATTATG  ldhD(LM)_fwd: TTGGGAGGGTATGAAGATTTTTGCTTACGG  ldhD(LM)_rev: CTTAAGGTCAAAAAAGCCGTGCGGCCGCTTAATATTCAACAGCAATAGCTG  ColE1+tra-F2: CCATCAAGAAGAGCGAC  pCD6-R1: GACTTTAAGC CTACGAATACC  seq_phA_ldhD_fwd: CTGAAAATAACGACACCG  seq_phA_ldhD_rev: GGCTTTTCATCATCACGA | This work.  *ldhD* (LEUM_ 1756) |
| pMTL83251_ P*_bgaL_*_NFP | -Plasmid-borne expression of codon-optimized *feg2-ldhD* fusion gene under the control  -Plasmid containing codon-optimized *feg2-ldhD* fusion gene under the control of the lactose-inducible P*_bgaL_* promoter and an erythromycin/ clarithromycin resistance gene *ermB* | N_FAST2_awo_opt_fwd: TTAAATGTATTGGGAGGGTGGATCCATGGAACAC GTTGCTG  N_FAST2_awo_opt_rev: CAAAGATCTTAGAACCACCACCACTACCCGTTTGACAAATACC  N_ldhD_awo_opt_fwd: CAAACGGGTAGGTGGTGGTGGTTCTAAGATCTTTGCTTATGGC  N_ldhD_awo_opt_rev: AGCTTGCATGTCTGCAGGCCTCGAGTTAATATTCAACCGCAATTG | Mook et al., 2022  GenBank-Nr. OL439953 |
| pMTL83251_ P*_lctA_*_NFP | -Plasmid-borne expression of codon-optimized *feg2-ldhD* fusion gene under the control  -Plasmid containing codon-optimized *feg2-ldhD* fusion gene under the control of the lactate-inducible P*_lctA_* promoter and an erythromycin/ clarithromycin resistance gene *ermB* | PlctA_fwd: ATTCGAGCTCGGTACCCGGGTCAGGACTTATCAAGTTTAAGT  PlctA_rev: CCATGGATCCACTCGCCCTCCATTAAATTAATTAAAG  N_FAST2_awo_opt_rev: CAAAGATCTTAGAACCACCACCACTACCCGTTTGACAAATACC  N_ldhD_awo_opt_fwd: CAAACGGGTAGGTGGTGGTGGTTCTAAGATCTTTGCTTATGGC  N_ldhD_awo_opt_rev: AGCTTGCATGTCTGCAGGCCTCGAGTTAATATTCAACCGCAATTG | Stock et al., 2025  GenBank-Nr. OL439953 |
| pMTL871ksb_P*_pta-ack_*_pflA | *-*Plasmid-borne expression of the *pflA* gene encoding a PFL-activating-enzyme from *C. pasteurianum*  -Plasmid containing *pflA* gene regulated by P*_pta-ack_* | P*_pta-ack_*_fwd: AGCGCCCAATACGCAGGGCCGGCGCCATCCTGCT TATTTGATTTACATTATATAATATTG  P*_pta-ack_*_rev: CCATTACCATGGTCCTCCCTTTAAATTTAAC  pflA_fwd: AGGGAGGACCATGGTAATGGGAAGAATTC  pflA_rev: TTTTTATCCTGCAGGGGGCCCTATTCTAATAATTCTTTTAAATATTTCTCAAG | *pflA* (AQ983_RS00460) |
| pMTL871ksb_P*_pta-ack_*_pflA_P*_ackA_*_-theo__pflB | *-*Plasmid-borne expression of the genes *pflB* and *pflA* encoding a PFL from *C. pasteurianum* and its activating enzyme  -Plasmid containing *pflA* gene regulated by P*_pta-ack_* and *pflB* gene regulated by P*_ackA_*_-theo_ and a chloramphenicol/ thiamphenicol resistance gene *catP* | P*_ackA_*_-theo__fwd: (AGCTCGGTACCCGGGATGGTATTATAGATATATAACTGTTATTATATAGAATACG)  P*_ackA_*_-theo__rev:  TTAAACAAGGATCCCTTGTTGCCCCTTCTCAG  pflB_fwd: AGGGGCAACAAGGGATCCTTGTTTAAACAATGGGAAGG  pflB_rev: GCATGTCTGCAGGCCCTCGAGTTATAATTTTTCATGAAAAGTTCTACTTATAAC | This work.  *pflA* (AQ983_ RS00460)  *pflB* (AQ983_ RS00455) |

**Description of *A. woodii ∆pyrE ∆lctBCD ∆pheA* and *A. woodii pheA_+_::ldhD***

The new recombinant strain *A. woodii ∆pyrE ∆lctBCD ∆pheA* (Figure S1 A-C) showed a phenylalanine auxotrophy, due to the knock-out of the *pheA* gene (Table 1).


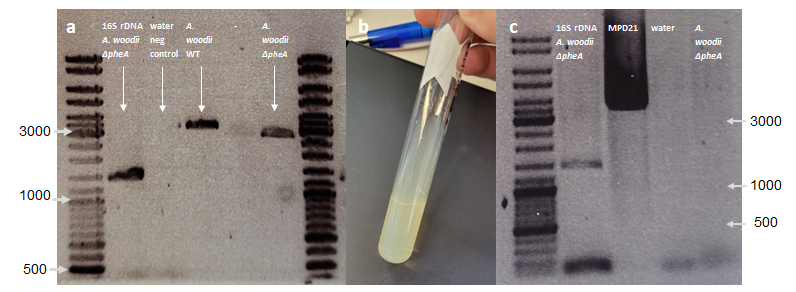


1 2 3 4

1 2 3 4 5

**Fig. S1.** Agarose gels showing DNA fragments obtained after PCR to verify *A. woodii ∆pyrE ∆lctBCD ∆pheA*. Slanted agar with *A. woodii ∆pyrE ∆lctBCD ∆pheA* growing on it. **a.** Marker: GeneRulerMix Thermo Fisher. Lane 1: Positive control, 16S rDNA (1500 bp) fragment, amplified from the gDNA isolated from *A. woodii* WT. Lane 2: Negative control (water). Lane 3: *pheA* gene fragment, from the gDNA isolated from *A. woodii* WT. Lane 4: Empty. Lane 5: *pheA* fragment amplified from the gDNA isolated from *A. woodii pheA::ldhD*.

**b.** Slanted YTF agar in a hungate with *A. woodii ΔpheA* colonies (exemplarily marked with arrows).

**c.** Marker: GeneRulerMix Thermo Fisher. Lane 1: Positive control, 16S rDNA (1500 bp) fragment, amplified from the gDNA isolated from *A. woodii* WT. Lane 2: Positive control, backbone (3000 bp) fragment, amplified from the template pMTL-MPD21 (expected fragment: 3000 bp), isolated from *E. coli* XL1-Blue [pMTL-MPD21]. Lane 3: Negative control (water). Lane 3: Negative control (water). Lane 4: No fragment, amplified from the gDNA isolated from *A. woodii pheA_+_::ldhD*.

Then the *pheA* gene was reintegrated in the genome of *A. woodii ∆pyrE ∆lctBCD ∆pheA* together with the *ldhD* gene, regulated by P*_bgaL_* (Figure S2 A, B, Table 1).


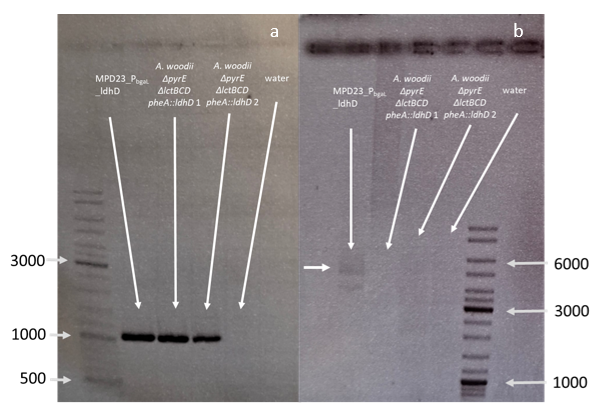


1 2 3 4

1 2 3 4

**Fig. S2.** Agarose gels showing DNA fragments obtained after PCR to verify *A. woodii pheA_+_::ldhD*.

**a.** Marker: GeneRulerMix Thermo Fisher. Lane 1: Positive control, fragment containing a part of the *pheA* gene, the P*_bgaL_* promoter, and a part of the *ldhD* gene (1513 bp), amplified from the template pMTL-MPD23_PbgaL_ldhD (expected fragment: 1513 bp), isolated from *E. coli* XL1-Blue [pMTL-MPD23_PbgaL_ldhD]. Lane 2 and 3: fragment containing a part of the *pheA* gene, the P*_bgaL_* promoter, and a part of the *ldhD* gene from the gDNA isolated from *A. woodii pheA::ldhD*. Lane 4: Negative control (water).

**b.** Marker: GeneRulerMix Thermo Fisher. Lane 1: Negative control, 5976 bp long fragment, amplified from the template pMTL-MPD23_PbgaL_ldhD (expected fragment: 5976 bp), isolated from *E. coli* XL1-Blue [pMTL-MPD23_PbgaL_ldhD]. Lane 2 and 3: no fragment, amplified from the gDNA isolated from *A. woodii pheA_+_::ldhD*. Lane 4: Control (water).

The new strain *A. woodii pheA_+_::ldhD* showed no phenylalanine auxotrophy anymore and was not resistant to thiamphenicol (Figure S3).


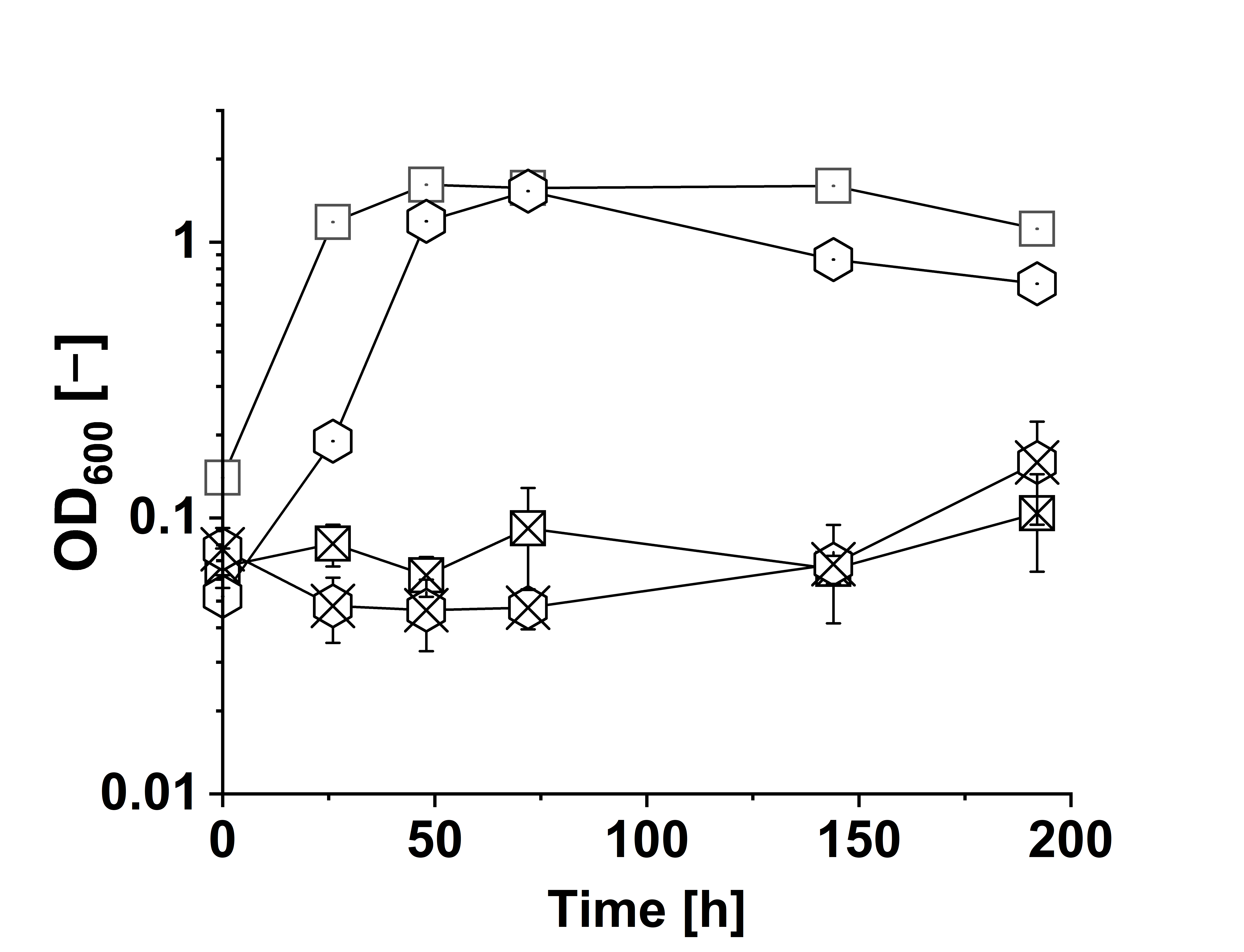


**Fig. S3.** Heterotrophic growth experiment (DSM 135 medium (Hoffmeister et al., 2016) without yeast extract, 40 mM fructose) with the strains *A. woodii* WT without thiamphenicol (white squares, n=1) *A. woodii* *pheA_+_::ldhD* without thiamphenicol (white hexagons, n=1), *A. woodii* WT with 15 µl thiamphenicol (crossed squares, n=3), *A. woodii* *pheA_+_::ldhD* with 15 µl thiamphenicol (crossed hexagons, n=3). OD_600_. Error bars indicate standard deviations.

*A. woodii pheA_+_::ldhD, A. woodii* P*_bgaL_*_LDHD, and *A. woodii* WT were cultivated in an autotrophic growth experiment. During this experiment, the *A. woodii* WT control reached its maximum OD_600_ of 1.73 ± 0.15. The maximum observed OD_600_ value of *A. woodii pheA_+_::ldhD* (not induced) was 1.58 ± 0.12. *A. woodii pheA_+_::ldhD* (induced) grew to a peak OD_600_ of 1.33 ± 0.14, and *A. woodii* P*_bgaL_*_LDHD induced to a peak OD_600_ of 0.84 ± 0.05 (Figure S4 A). Furthermore, the extracellular pH value was maintained between 6 and 7.5 (Figure S4 B). *A. woodii* WT recorded the highest total pressure loss of 4833 ± 225 hPa in the experiment. Followed by *A. woodii pheA_+_::ldhD* (induced) with a pressure loss of 4767 ± 189 hPa during the cultivation.

*A. woodii pheA_+_::ldhD* (not induced) listed a pressure decline of 4683 ± 339 hPa H_2_ and CO_2_. In contrast, *A. woodii* P*_bgaL_*_LDHD (induced) accumulated a total pressure decline of 1840 ± 43 hPa (Figure S4 C).

Under these conditions, *A. woodii* P*_bgaL_*_LDHD (induced) produced a maximum of 9.09 ± 0.42 mM lactate, thereof, 8.6 mM was produced post-induction. Normalized to an OD_600_ of 1, this corresponds to a maximum post-induction lactate concentration of 14.21 ± 0.84 mM OD600. *A. woodii* *pheA_+_::ldhD* (induced) produced after 168 h, a maximum lactate concentration of 2.59 ± 0.22 mM was produced, all post-induction. This corresponds to a post-induction lactate concentration of 2.0 mM, normalized to an OD_600_ of 1 (Table S1, Figure S4 D). *A. woodii* *pheA_+_::ldhD* (not induced) and *A. woodii* WT produced no lactate.

Another product was formate, as reported previously by Mook et al. (2022). *A. woodii* P*_bgaL_*_LDHD (induced) produced a maximum formate concentration of 19.83 ± 4.89 mM (44.52 ± 3.95 mM OD600_600_) after 168 h, while *A. woodii pheA_+_::ldhD* (induced) produced 6.90 ± 1.4 mM formate (5.75 ± 2.14 mM OD600_600_) after 168h. *A. woodii* WT and *A. woodii pheA_+_::ldhD* (not induced) produced formate concentrations of 4.90 ± 2.24 mM (3.33 ± 1.73 mM OD600_600_) and 1.78 ± 0.62 mM formate (1.32 ± 0.55 mM OD600_600_) after 168 h, respectively (Figure S4 E). The maximum post-induction lactate:formate ratio of *A. woodii* P*_bgaL_*_LDHD and *A. woodii pheA_+_::ldhD* induced were 0.46 and 0.37 (Table S2).

The main product of all investigated *A. woodii* strains was acetate. *A. woodii* P*_bgaL_*_LDHD produced 159 ± 5.6 mM acetate after 168 h, while *A. woodii* *pheA_+_::ldhD* (induced) produced 326.1 ± 17.2 mM acetate after 88 h (Figure S4 F). The maximum post-induction lactate:acetate ratio was 0.06 for *A. woodii* P*_bgaL_*_LDHD and 0.01 for *A. woodii* *pheA_+_::ldhD* induced (Table S2).


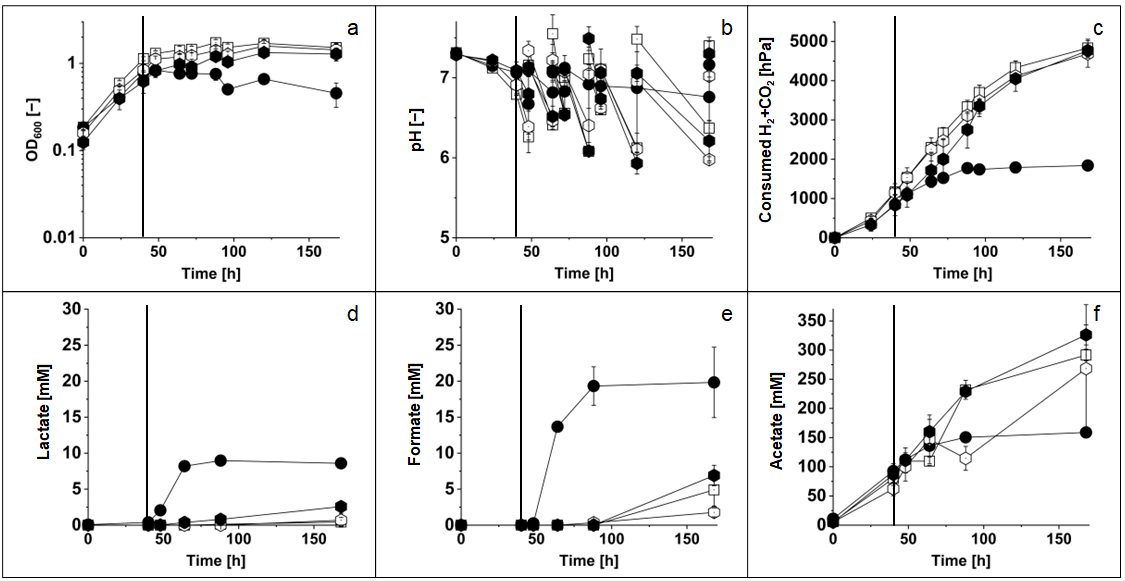


**Fig. S4.** Autotrophic growth experiment with the strains *A. woodii* WT (white squares), *A. woodii* P*_bgaL_*_LDHD induced (black circles), *A. woodii* *pheA_+_::ldhD* not induced (white hexagons), *A. woodii* *pheA_+_::ldhD* induced (black hexagons). OD_600_ (**a**), pH (**b**), consumption of H_2_ and CO_2_ (**c**), lactate concentration (**d**), formate concentration (**e**), and acetate concentration (**f**) were monitored. The dotted line shows the induction of the *A. woodii* P*_bgaL_*_LDHD cells with 2.5 mM lactose. The full line shows the induction of the *A. woodii* P*_bgaL_*_LDHD_PFL cells with 2.5 mM lactose and 1 mM theophylline. n = 3. Error bars indicate standard deviations.

**Table S2.** Maximum D-lactate concentration post-induction, maximum molar D-lactate:acetate ratio post-induction, maximum OD_600_ specific D-lactate concentration post-induction, and maximum D-lactate production rates post-induction.

|  | ***A. woodii* P*_bgaL_*_LDHD** | ***A. woodii pheA_+_::ldhD*** |
| --- | --- | --- |
| Maximum D-lactate concentration post-induction [mM] | 9.09 ± 0.42 | 2.59 ± 0.22 |
| Maximum molar D-lactate/acetate ratio post-induction [−] | 0.06 ± 0.00 | 0.01 ± 0.00 |
| Maximum produced D-lactate concentration post-induction normalized to OD_600_ [mM OD_600_^-1^] | 14.21 ± 0.84 | 2.11 ± 0.52 |
| Maximum D-lactate production rate post-induction [mM · h^-1^] | 0.33 ± 0.02 | 0.02 ± 0.00 |
